# Exploring the potential of halotolerant bacteria from coastal regions to mitigate salinity stress in wheat: physiological, molecular, and biochemical insights

**DOI:** 10.3389/fpls.2023.1224731

**Published:** 2023-09-22

**Authors:** Muhammad Aizaz, Lubna, Waqar Ahmad, Ibrahim Khan, Sajjad Asaf, Saqib Bilal, Rahmatullah Jan, Saleem Asif, Muhammad Waqas, Abdul Latif Khan, Kyung-Min Kim, Ahmed AL-Harrasi

**Affiliations:** ^1^ Natural and Medical Science Research Center, University of Nizwa, Nizwa, Oman; ^2^ Department of Engineering Technology, University of Houston, Sugar Land, TX, United States; ^3^ Department of Applied Biosciences, Kyungpook National University, Daegu, Republic of Korea; ^4^ Department of Agriculture Extension, Government of Khyber Pakhtunkhwa, Mardan, Pakistan

**Keywords:** halotolerant, bacteria, seawater, salinity, alleviation, abiotic stress

## Abstract

Salinity stress, a significant global abiotic stress, is caused by various factors such as irrigation with saline water, fertilizer overuse, and drought conditions, resulting in reduced agricultural production and sustainability. In this study, we investigated the use of halotolerant bacteria from coastal regions characterized by high salinity as a solution to address the major environmental challenge of salinity stress. To identify effective microbial strains, we isolated and characterized 81 halophilic bacteria from various sources, such as plants, rhizosphere, algae, lichen, sea sediments, and sea water. We screened these bacterial strains for their plant growth-promoting activities, such as indole acetic acid (IAA), phosphate solubilization, and siderophore production. Similarly, the evaluation of bacterial isolates through bioassay revealed that approximately 22% of the endophytic isolates and 14% of rhizospheric isolates exhibited a favorable influence on seed germination and seedling growth. Among the tested isolates, GREB3, GRRB3, and SPSB2 displayed a significant improvement in all growth parameters compared to the control. As a result, these three isolates were utilized to evaluate their efficacy in alleviating the negative impacts of salt stress (150 mM, 300 mM, and seawater (SW)) on the growth of wheat plants. The result showed that shoot length significantly increased in plants inoculated with bacterial isolates up to 15% (GREB3), 16% (GRRB3), and 24% (SPSB2), respectively, compared to the control. The SPSB2 strain was particularly effective in promoting plant growth and alleviating salt stress. All the isolates exhibited a more promotory effect on root length than shoot length. Under salt stress conditions, the GRRB3 strain significantly impacted root length, leading to a boost of up to 6%, 5%, and 3.8% at 150 mM, 300 mM, and seawater stress levels, respectively. The bacterial isolates also positively impacted the plant’s secondary metabolites and antioxidant enzymes. The study also identified the *WDREB*2 gene as highly upregulated under salt stress, whereas *DREB*6 was downregulated. These findings demonstrate the potential of beneficial microbes as a sustainable approach to mitigate salinity stress in agriculture.

## Introduction

The increase in human population is directly proportional to environmental damages resulting from growing industrialization, which led to decreased agricultural land. Feeding the growing world population in the next ten to twenty years will be challenging (Steensland and Zeigler, 2021; Soria-Lopez et al., 2022). Numerous biotic and abiotic stresses significantly impact plant development, productivity, yield, and food quality (Shi-Ying et al., 2018; Chaudhry and Sidhu, 2022). The biotic stresses consist of several pests or pathogens that cause infections or injuries. Drought, salinity, heat, heavy metals, and other organic pollutants are examples of abiotic stresses. Soil salinization is the most harmful of all abiotic stresses and is regarded as one of the major factors limiting agricultural output and food security (Daliakopoulos et al., 2016). Salinity affects 20% or more of the world’s agricultural area, and this percentage is rising continuously (Hopmans et al., 2021). By 2050, it is estimated that about 50% or more of agricultural land will be damaged by salinity. The leading cause of salinization of agricultural land is the deposition of salts in the soil (Corwin, 2021; Hassani et al., 2021), mainly sodium (Na+) and chloride (Cl-) ions. Water conductivity, soil porosity, and aeration are all inhibited by high Na+ accumulation. Furthermore, soil salinity stress adversely affects microbial diversity in and around plant roots (Hou et al., 2021). A plant under salinity stress experiences several morphological, physiological, and molecular alterations that hinder its ability to grow and develop.

For example, the rate of photosynthesis, stomatal conductance, and enzyme activity are all impacted by excessive salt concentrations (Jangra et al., 2022; Aizaz et al., 2023). Salt stress boosts reactive oxygen species (ROS) by causing oxidative stress, damaging cell membranes, proteins, lipids, and nucleic acids (DNA, RNA), and may also cause programmed cell death (Zhang et al., 2016). Due to the excessive accumulation of Na+ and Cl- ions, salinity also causes hypertonic stress (Shi-Ying et al., 2018). Various approaches are utilized to improve crop resilience to salt stress, encompassing breeding, genetic engineering, CRISPR/Cas9 technology, chemical priming, and biological priming (Godoy et al., 2021; Wang et al., 2023). In order to mitigate the adverse impacts of salt stress on plants, the utilization of salt-tolerant bacteria presents a promising solution. These remarkable microorganisms possess the ability to adhere to plant roots, maintaining their presence and effectively countering the harmful effects of high salt levels. Through their biocontrol capabilities, they contribute to fostering healthier growth in plants. As a result, rooting processes are significantly enhanced, leading to a substantial boost in crop yields of up to 10–15% (Rima et al., 2018).

Furthermore, utilizing Plant Growth-Promoting Bacteria (PGPB) proves to be a cost-effective and highly efficient approach to addressing the challenges posed by developing new salt-tolerant plants. This choice becomes even more compelling due to the complexities of understanding abiotic stress tolerance mechanisms, growing awareness of agrochemical toxicity, and emerging alternative eco-friendly technologies. As a result, PGPB emerges as a promising solution to boost plant resilience to salt stress while bypassing the difficulties associated with traditional methods of developing tolerant plants (Mohammadipanah and Zamanzadeh, 2019; Alberton et al., 2020; Jiang et al., 2021). PGPB endophytes inhabit healthy plant tissues without harmful effects. These endophytic PGPB can improve plant development and resistance to saline stress (Lu et al., 2021). PGPB are typically found in saline soil at the root zone of plants and mediate some of the mechanisms like biofilm formation, extracellular polymeric substance (EPS) production, nitrogen fixation, phytohormone production, and ACC-deaminase activity (Ansari et al., 2019). Further, PGPB stimulates antioxidant activity during salt stress and encourages plant nutrient uptake and homeostasis (Kumar et al., 2020).

Some PGPBs can alleviate the adverse negative effects on plants caused by salinity and promote growth by producing phytohormones. Plant behavior under salinity stress is influenced by the phytohormones produced by bacteria because, under some circumstances, plants do not produce enough phytohormones for proper development (Verma et al., 2021; Haile et al., 2023). A halotolerant strain *Kocuria rhizophila* Y1, isolated from the rhizosphere of maize, can withstand up to 10% NaCl and protect maize plants against salt stress by modulating plant hormone (IAA and ABA) levels and enhancing nutrient uptake (Li et al., 2020). Similarly, wheat plants treated with different PGPBs like *Bacillus* sp., *Azospirillum brasilense*, *Azospirillum liprum*, and *Pseudomonas stutzeri* as a consortium resulted in increased plant biomass and relative water content via producing different phytohormones. Using the appropriate features that distinguish them as plant-growth promoters, soil bacteria can enhance plant nutritional status under salt stress in various ways (Franchi and Fusini, 2021; Gao et al., 2022). Salt stress led to a significant reduction in the biomass of wheat plants, with the decline being more pronounced under high salt stress conditions compared to lower stress levels (Radi et al., 2013). Wheat exhibits a relative tolerance to salt with a value of 7.0 dS·m^−1^. However, as the salt concentration in the soil increases to 9.0 dS·m^−1^, the yield of wheat experiences a decline of approximately 25%. These results indicate that wheat can endure moderate levels of salinity, but if the salt content in the soil becomes too high, it adversely affects its productivity, resulting in reduced yields (Galvan-Ampudia and Testerink, 2011). The reduction in growth and yield varies between cultivars and salt concentrations of the medium (Sultana et al., 1999).

Research has extensively documented the application of PGPR to mitigate abiotic stresses in plants, leading to a significant boost in sustainable agriculture (Hadi et al., 2011; Ayaz et al., 2021). One promising area of study revolves around the use of PGPR to enhance plant growth in the face of salt stress, making it an emerging and innovative technology (Grover et al., 2011). Numerous researchers are actively exploring the potential of PGPR in alleviating salt stress for various crops, showcasing the growing interest in this field (Ansari et al., 2019). The interaction between microorganisms and plants offers a range of intricate mechanisms that help plants develop resistance to salt stress (Smith et al., 2017). Past research has shown that certain beneficial plant growth-promoting rhizobacteria (PGPR), such as *Pesudomonas* spp. and *Bacillus* spp., isolated from saline soil, can enhance plant growth even in the presence of high salinity (Ali et al., 2022b). This intriguing concept has captured the attention of researchers, leading them to explore effective biotechnological approaches that involve utilizing PGPR as a valuable resource to mitigate the adverse effects of salinity on plants and ultimately boost their growth under salt stress (Khan et al., 2019; Ali et al., 2022b). In recent times, noteworthy advancements have been made in utilizing PGPR to mitigate salt stress in wheat plants. This has improved growth and productivity in saline regions (Smith et al., 2017). PGPR, along with specific compounds, has shown the ability to enhance the plant’s ability to withstand salt stress by influencing hormonal, photosynthetic, and ROS scavenging pathways (Bharti et al., 2014; Sousa et al., 2021). Because salt changes the balance of ions in saline soils, the bioavailability of nutritional components is poor (Mohanapriya et al., 2022). However, halotolerant PGPB can reduce some of the adverse effects of salinity to a certain extent, enhancing plant growth, biomass accumulation, and yield (Munns and Tester, 2008; Khan et al., 2021). When tomato plants were exposed to salinity, *Achromobacter piechaudii* ARV8 improved P uptake and water balance, which had a favorable effect on the plants’ growth (Mayak et al., 2004). *Arthrobacter, Bacillus*, *Beijerinckia*, *Burkholderia*, *Enterobacter*, *Pseudomonas*, *Erwinia*, *Mesorhizobium*, *Flavobacterium*, *Rhodococcus*, and *Klebsiella* are some of the phosphate-solubilizing PGPB (Mitra et al., 2020; Kaur et al., 2021; Amri et al., 2022; Belkebla et al., 2022). Among these bacteria, some may fix soluble P in their cells or, by using different phosphatases, convert P from organic to inorganic forms (Noori et al., 2018; Etesami, 2020). The microbial cell secretes a variety of biopolymers into its environment, including polysaccharides, polyesters, and polyamides. In plant-microorganism interactions, particularly in reducing plant salt stress, the bipolymers perform a crucial and indispensable role (Lastochkina et al., 2021; Saberi Riseh et al., 2021; Krishnamoorthy et al., 2022). The potential benefits of PGPB for promoting plant development are immense, and the global bio-inoculant market is expected to expand at about 10% annually (Vassileva et al., 2021).

This study aimed to isolate halotolerant bacteria from coastal regions characterized by high salinity, including various sources such as plants, rhizosphere, algae, lichen, sea sediments, and sea water. The primary objective was to characterize these bacteria for their potential to promote plant growth under normal and salt stress conditions, specifically by evaluating their ability to solubilize phosphate, produce siderophores, and synthesize indole-3-acetic acid (IAA). Through this process, the most effective bacterial strains for promoting plant growth were identified and subsequently inoculated into wheat plants subjected to different levels of salt stress to evaluate the interaction between the strains and the plants. In conclusion, these bacterial strains can be used practically in sustainable agriculture as biofertilizers or bioinoculants to enhance plant growth and improve crop yield in saline-affected areas.

## Methodology

### Isolation of bacteria

Bacteria were isolated from different sources, such as plants, rhizosphere, algae, lichen, sea sediments, and sea water, using appropriate methods that were previously described, with minor adjustments made to the procedures.

### Isolation of endophytes bacteria

Four plants (*Tetraena qaterensis*, *Suaeda aegyptiaca*, *Paspalum vaginatum*, and *Avicennia marina*) located in the coastal region were chosen to explore the diversity of culturable bacterial endophytes. The plant materials were carefully washed to remove all soil particles and debris. The plant was surface sterilized in 75% ethanol next to this 2% Sodium hypochlorite for 1 minute and then rinsed with sterile distilled water twice. A last rinse with sterile water was performed before testing the efficiency of the sterilization process in order to exclude non-endophytes. After sterilization, the plant material was allowed to dry on a sterile filter paper; the plant parts were cut into small pieces of about 3-3.5 cm through a sterile scalpel. Four pieces of every part were placed on plates containing Luria-Bertani (LB) agar medium supplemented with 300 mM and 600 mM NaCl and incubated for six days at 26-30°C. The bacterial endophyte growing in the form of spots or layers in plant tissues were picked up and subcultured on LB agar medium using a sterilized loop. The bacterial colony was purified via sub-culturing until pure strain was obtained on LB agar medium.

### Isolation of bacteria from plant rhizosphere

The Volume Displacement Technique was used to isolate the bacterial strains from the rhizosphere (Lorenz et al., 1994). This technique involved placing a 2 cm piece of root and the attached soil in 90 mL of sterile distilled water-containing flasks, shaking the flasks for 15 minutes, and then lifting the roots. This process was repeated, and roots were added until the total volume of soil and water was 100 mL (Al-Abbasi et al., 2021). About 1000-fold dilutions were prepared, and 30 μL was spread on the plates containing Luria-Bertani (LB) agar medium supplemented with 300 mM and 600 mM NaCl and kept in an incubator at 30 ± 1°C.

### Isolation of bacteria from algae

In order to isolate bacterial endophytes from algae samples, the algae were first washed with freshwater and then subjected to surface sterilization, as reported previously (Deutsch et al., 2021). This was achieved by dipping the algal pieces twice for 3 seconds each in 70% ethanol, followed by two 1-minute washes in distilled water. The effectiveness of the sterilization procedure was confirmed by inoculating appropriate agar media with the water from the final wash and observing the absence of bacterial growth. The surface-sterilized algae were then cut into small pieces (0.5-1 cm) and placed on nutrient agar (NA) growth media in 90-mm petri plates. At least two plates with 10 pieces each were used for endophyte isolation and collection for each type of algae. The plates were incubated at 27°C for 8 days to allow for the growth of endophytes out of the algal pieces. The resulting bacterial colonies were then collected and reinoculated on appropriate growth media to ensure the purity of the cultures. A single colony was established for each endophyte.

### Isolation of bacteria from lichen

In order to isolate bacteria from lichen, we followed the protocol outlined by (González et al., 2005). Briefly, each lichen sample (300-500 mg) was surface washed twice with sterile water, homogenized with 30 mL of sterile water using a blender, and subjected to serial dilutions that were plated onto selective actinomycete isolation media (including soil extract agar, humic acid agar, and glycerol asparagine agar) amended with cycloheximide (80 μg/mL) and nalidixic acid (20 μg/mL). Alternatively, the lichen samples were subjected to dry heat at 100°C for one hour prior to homogenization and plating. Individual colonies were then isolated and grown on YME agar medium (containing 0.4% yeast extract, 1% malt extract, 0.4% glucose, and 0.2% Bacto-agar) at 28°C.

### Isolation of bacteria from sea sediments

In order to isolate halophilic bacteria, we followed the protocol outlined by (Guan et al., 2020). Sediments weighing 10 grams were dispersed into 90 mL of sterilized NaCl brine (5% or 15%, w/v) and incubated at 37°C for 60 minutes with shaking at 200 rpm. The resulting slurry was then serially diluted with sterilized NaCl brine (5% or 15%, w/v), and aliquots of each dilution (0.1 mL) were spread onto Petri dishes using nine different media types (**Table 1**) to isolate bacteria. To ensure accuracy, each type of medium plate was replicated three times to calculate the number of colonies. All agar plates were supplemented with 5% or 15% (w/v) NaCl, and nystatin (50 mg·L-1) was added to suppress the growth of non-bacterial fungi. The Petri dishes were then incubated at 37°C for one to six days Colonies were picked based on their size and color and further purified on inorganic salts-starch agar or TSA supplemented with 5% or 15% (w/v) NaCl. The purified colonies were stored as glycerol suspensions (20%, v/v) at -20°C, or as lyophilized cells for long-term storage at -4°C.

**Table 1:** Isolation of bacterial isolates from 5 different locations in Muscat, Oman, from different sources.

Sources	Code	Number of isolates	EC (mS/cm)	pH
*Tetraena qaterensis*	TQ	18	4.6 ± 0.02	7.8 ± 0.02
*Suaeda aegyptiaca*	SA	8	6.2 ± 0.065	8.01 ± 0.03
Sand inside water (sea sediment)	SD	19	6.8 ± 0.04	8.1 ± 0.02
*Paspalum vaginatum* (Grass)	GR	10	3.2 ± 0.03	7.8 ± 0.01
*Avicennia marina* (Mangroves)	MG	7	2.4 ± 0.07	7.5 ± 0.4
Lichens	LN	4	2.3 ± 0.06	7.6 ± 0.02
Algea	SP	10	45.5 ± 0.36	7.3 ± 0.04
Sea water 1	1W	2	49.33 ± 0.45	9.03 ± 0.03
Sea water 2	2W	3	50.23 ± 0.56	8.15 ± 0.04

The abbreviation used with sources in bacterial strains means EB, endophytic bacteria; RB, rhizospheric bacteria; SB, sand or soil bacteria near source; WB, water bacteria.

### Isolation of bacteria from soil

In this study, we followed the protocol described by Prashanthi and GK (2021) to collect soil samples using sterile spatulas, located about 1 foot away from the root zone to differentiate from rhizospheric soil. Soil bacteria were isolated via serial dilution on nutrient agar as per MMM’s protocol. Suspensions of 1 gram soil in 10 ml distilled water underwent dilution from 10^-1^ to 10^-6^. The spread plate technique isolated organisms: 0.1 ml diluted sample pipetted on nutrient agar, spread, and incubated at 37°C for 24 hours. Prominent colonies were isolated, stored at 4°C.

### Isolation of bacteria from seawater

Two seawater samples collected from two different places were subjected to serial dilution to isolate bacteria from seawater. Around 10 mL of seawater sample was mixed with 90 mL of sterile distilled water in a 250 mL flask to obtain 10^-1^. Similarly, 1 mL from this dilution was taken and added to another 9 mL of sterile distilled water in test tubes from 10^-2^ and repeated once similarly to get a 10^-3^ dilution. Around 0.1 mL from the 10^-3^ dilution was used to spread the LB medium Petri plates supplemented with 600 mM NaCl. The plates were then incubated for 24 hours, and the appearance of bacterial colonies were observed. Distinct bacterial colonies were isolated based on morphological characteristics and stored as glycerol stocks at -20°C.

### Electrical conductivity and pH of the samples

The methodology described by Delgado-García et al. (2018) was used to prepare saturation extracts of soil and sand samples. This involved mixing 250 g of soil with distilled water and allowing it to rest for four hours, followed by obtaining the saturation extract through filtration and centrifugation (6000 rpm, 10 minutes at room temperature). Each soil extract’s electrical conductivity (EC) was measured using a conductivity meter (inoLabÒ Multi720 WTW, Germany) at room temperature. For the seawater sample, the same procedure was followed without the need for the preparation of a saturation extract.

### Screening bacterial strains for IAA production

An initial assessment was performed to test the phytohormone production potential of bacteria by adding 1 mL of Salkowski reagent to 2 mL of culture filtrate. The colorimetric approach was used to screen these bacterial strains for IAA synthesis (Lubna et al., 2020). The bacterial strains were cultured in an incubator at 30°C in LB broth medium. The samples were filtered after seven days, and the IAA concentrations in the culture filtrates were determined by adding 1 mL of Salkowski reagent to 2 mL of each culture filtrate, followed by 30 minutes of incubation in the dark. The optical density was measured at 530 nm using UV spectrophotometer. The amount of IAA produced was calculated by the standard graph of pure indole acetic acid (Gordon and Weber, 1951). IAA-producing strains were chosen for future study.

### Quantitative estimation of phosphate solubilization bacteria

The vanado-molybdat phosphoric method (Murphy and Riley, 1962) was employed to quantitatively estimate P solubilization. Specifically, a 50 mL volume of NBRIP broth medium was prepared in a 250 mL flask, with pH adjusted to 7.0 ± 0.2 prior to autoclaving. Subsequently, a fresh inoculum of 200 μl was introduced, and the flask was subjected to shaking conditions at 120 rpm/min and 28°C for 7 days. As a control, NBRIP broth medium that was both autoclaved and uninoculated was utilized. Following incubation, 2 mL of the culture was centrifuged at 10,000 rpm for 15 min, and the soluble P content was assessed through colorimetry using the molybdenum blue method. Optical density was measured via a UV–VIS spectrophotometer at 430 nm. All experimental conditions were performed in triplicate.

### Siderophore production

Siderophore production of each bacterial isolate was determined using the Chrome-Azurol S (CAS) medium and the Universal Chemical Assay (Senthilkumar et al., 2021). The experiments were conducted in four replicates to ensure the accuracy and consistency of the results. Briefly, bacterial strains were spotted onto CAS plates and incubated for four days at 28°C. The presence of an orange halo around the colonies indicated the production of siderophore.

### Screening bacterial strain on wheat seed germination

All 81 isolated bacterial strains were inoculated to wheat seeds to evaluate their effect on germination and seedling growth in normal conditions. Seeds of a disease-resistant and high-yielding wheat cultivar (Akbar-19; Lot No. KL-690601) were acquired from the KPK Agriculture Research Center in Pakistan and assessed for viability before use. Surface seed sterilization was done with sodium hypochlorite (Sigma Aldrich, St. Louis, MO, USA), rinsed with sterile distilled water more than three times, and dried through sterile filter paper. Furthermore, wheat seeds were soaked in 100 mL bacterial culture suspension for 5 h. Bio-priming of seed was conducted using the abovementioned bacterial culture filtrate for 5 h at 25°C under dark conditions. After 5 hours, the seeds were washed carefully with distilled water and dried for 72 hours at room temperature with the help of sterile filter paper (Mitelut and Popa, 2011). A germination test was carried out to obtain inoculated wheat seed quality under optimal conditions. The seeds were sown in 20 cm diameter plates with sterile filter paper, and each replica consisted of 20 seeds (3 replicas for each strain). The Petri plates were then placed into the germination chamber at 25°C. Each petri plate was given 5 mL of distilled water as required, and the germination percentage was measured 24 hours for 10 days. After 14 days of seed germination, the length of the shoot and roots were measured with a ruler. An analytical balance was used to determine ten seedlings’ fresh shoots and root weight. Three bacterial isolates including GREB3, GRRB3, and SPSB2, were selected for further experiments based on their effect on wheat seedling germination and seedling growth.

### Bacterial strain’s interaction with wheat plants under Salt stress

Wheat seeds were acquired from the KPK Agriculture Research Center in Pakistan and assessed for viability before use. The abovementioned technique was used to sterilize the seeds (Lubna et al., 2020). Seeds were germinated in germination trays for ten days to produce uniform plants. The sterilized germination trays and pots were filled with sterile horticulture soil. The horticulture soil composition was as described by (Lubna et al., 2020). After 14 days, randomly selected uniform wheat seedlings were planted, five plants per plastic pot (10 × 9 cm). Bases were utilized to prevent pollution from irrigation water leaching. The experimental design: was contained (a) Control (untreated plants), (b) Inoculated plants with three bacterial strains (each strain was applied separately), (c) Plants treated with 150 mM (NaCl), (d) Plants treated with 300 mM (NaCl), (e) Plants treated with SW (seawater) treatment, (f) Plants treated with respective bacterial strain + 150 mM (NaCl), (g) Plants treated with respective bacterial strain + 300 mM (NaCl) and (h) Plants treated with respective bacterial strain + SW (seawater). The growth chamber conditions were as follows: day/night cycle 14 h at 28°C/10 h at 25°C and 60–70% relative humidity. In the current experiments, we used seawater and two NaCl dilutions (150 mM and 300 mM) to irrigate their respective pots. Bacterial inoculation was carried out on the 3rd, 7th, and 14th days after transplanting the wheat seeds. Bacterial strains were allowed to grow in NB media until OD600 reached 0.8. The grown culture was centrifuged at 8000 rpm for 10 min at 4°C, and cell pellets were re-suspended in distilled water. The absorbance was adjusted to an optical density of 0.8 at 600 nm (cell count ~ 1.0 × 108 CFU mL^−1^). Fifteen mL (15 mL) bacterial suspension was applied near the root zone. The un-inoculated control plants were treated with only distilled water at the time of inoculation. Salt stress was applied at two different levels, 150 mM NaCl, 300 mM NaCl and seawater (SW). Briefly, 21-day-old wheat seedlings were treated with 15 mL of 150 mM NaCl, 300 mM NaCl, and SW near the root zone, and the salt treatment was applied three times at three days intervals. Plants were harvested after 15 days of salt stress imposition. After measuring shoot and root length and their fresh biomass, the plants shoots and roots were separated and promptly stored in liquid nitrogen for RNA extraction and antioxidant analysis, then kept at -80°C for later use.

### Determination of protein and catalase activity

Protein contents were determined by following Bradford (Bradford, 1976) protocol. Fresh leaf ground in liquid nitrogen, used 1 mL sodium phosphate buffer (100 mM, PH 7) including ethylene diamine tetra acetic acid (EDTA: 1mM), MgCl_2_ (3mM), PVP (2%), and Tris 50 mM and 10 minutes centrifuged at (10000× g) to obtain the resultant crude mixture (150 μL supernatant, 150 μL distilled water and 300 μL Bradford regent). The sample was calculated at an absorption value of 595 nm. The sample extract was quickly combined with (0.2 M) H_2_O_2_ in 10 mM Calcium Phosphate Buffer (PH: 7) using an analyzed spectrometer at an absorbance of 240 nm.

### Determination of total polyphenol, polyphenol oxidase, flavonoid, and flavanol activity

The total polyphenolic content of fresh leaves was determined using the Folin-Ciocalteu method. Initially, 200 mg of the leaves were ground in liquid nitrogen and mixed with 1 mL of 80% ethanol. The mixture was then centrifuged for 5 minutes at 10,000 rpm, and 500 μL of the resulting extract (after centrifugation) was combined with 500 μL of Folin-Ciocalteu reagent. Subsequently, 500 μL of 10% Na2CO3 was added to the mixture, which was then left to stand in the dark at room temperature for 1 hour. Following this, the mixture was centrifuged for 15 minutes at 15,000 rpm, and the supernatant was collected for analysis. Using the spectrophotometer, the absorbance of the standard and samples were determined at 750 nm (Haghighi and Saharkhiz, 2021). The PPO assay was carried out according to previous studies (Kumar and PA, 1982). The PPO assay solution contained 2 mL of 0.1 M phosphate buffer having pH 6.0, 1 mL of 0.1 M catechol, and 0.5 mL of enzyme extract. The resultant sample mixture was incubated at ambient temperature for 5 minutes. The reaction was stopped by adding 1 mL of 2.5 N solution of H_2_SO_4_. It was observed that purpurogallin gave absorbance at 495 nm. Blank was obtained using the same assay mixture by adding 2.5N H_2_SO_4_ without further incubation. TPP and PPO activity is articulated in U/mg protein. According to the total flavonoid, methanolic extracts were assessed, giving an absorption peak at 510 nm. In order to determine the total flavonoids, Catechin was taken as a standard (Taban et al., 2021). Furthermore, flavonols were determined by macerating dried powdered roots (0.5 g) in 3 mL ethyl alcohol (80%) for 24 hours at RTP. The filter paper was used to filter the resulting suspension. By combining 1 mL of aluminum chloride (2%) in ethanol (95%), a resulting solution (1 mL) was obtained. After 20 minutes, the optical density of the combination was estimated at 390 nm (Levon and Klymenko, 2021).

### RNA isolation and qRT-PCR

We randomLy collected leaves from each set of wheat plants following salt stress to measure the expression levels of the *DREB2*, *WDREB2*, and *DREB6* genes. The leaves were frozen in liquid nitrogen and kept at 80°C for subsequent stress analysis. To extract RNA and construct cDNA, an optimized protocol was adopted with minor modifications (Liu et al., 2018). Tris-HCL (0.025 M, pH: 7.5) was produced with 1% w/v SDS, 20 mM EDTA, 0.25 M sodium chloride, and 4% w/v Polyvinyl Pyrrolidone. The powdered plant (100 mg) was carefully transferred to 2 mL RNase-free microtubes containing extraction buffer (750 µL). Then, in an equal amount, blend it with chloroform isoamyl alcohol (CI; 24:1 v/v). In each tube, an equal volume of PCI (phenol:chloroform: isoamyl alcohol; 25:24:1 v/v) was mixed with the supernatant. The resulting mixes were smoothly shacked and centrifuged (12,000 g, 4°C for 10 minutes). After that, the upper (clean and clear) layer was transferred to a (1.5 mL) microcentrifuge tube, and 1/10 volume of sodium acetate (3 M, pH 5.2) was applied. Finally, all the samples were centrifuged again (12,000 g, 4°C for 10 minutes), and the pellet was cleaned with 75% pure ethanol. After 5 minutes of air drying, the pellet was dissolved in 50 µL of TE buffer. The RNA was then tested using NanoDrop and the Qubit broad-range kit (3.0), and the quality was confirmed using gel electrophoresis. In PCR tubes, 10 µL (>100 ng/L) of extracted RNA and an initially prepared Master Mix (RT buffer (2 µL), 25x dNTPs (0.8 µL), random primers (2 µL), reverse transcriptase (1 µL), and nuclease-free water (3.2 µL) were used to synthesize cDNA. The PCR reaction was cycled through a thermos cycler at 25°C for 10 minutes, 37°C for 2 hours, and 85°C for 5 minutes, with the temperature adjusted accordingly. When the reaction was finished, the cDNA was quantified using a Qubit DNA broad-range kit and kept at 80°C for molecular analysis. The primer sequence and accession number of each gene are shown in **Supplementary Table 1**. The amplified cDNA was used to perform qRT-PCR to determine stress-related genes relative expression. The actin gene was used as a reference gene. Power SYBR Green Master Mix and primers (forward and reverse 10 pM) were used in a thermocycler to perform PCR reactions for all genes of interest. The reaction was done three times for each sample to reduce the experimental error. The following PCR conditions were used: 10 minutes at 94°C, followed by 35 cycles at 94°C (45 s), 65°C (45 s), and 72°C (1 min), with the extension step at 72°C (10 min). The gene amplification threshold was set at 0.1. Each sample was run three times with three different replicates.

### Bacterial identification and phylogenetic analysis

Genomic DNA was extracted from endophytic bacterial cultures through enzymatic hydrolysis (Saito and Miura, 1963). The complete 16S rRNA gene (1.4–1.5 kb) was amplified via PCR, using universal bacterial primers 27 F (5′-AGA GTT TGA TCC TGG CTC AG-3′) and 1541R (5′-AAG GAG GTG ATC CAG CCG CA-3′). The amplification was carried out on a DNA engine gradient thermal cycler (BIO-RAD, USA). The 50 μL PCR reactions contained 4 μL of 2.5 U/μLTaq DNA polymerase (Tiangen, Beijing), 5 μL of 10× buffer (Tiangen), 1 μL of 20 mM dNTPs (Tiangen), 37 μL of SDW, 1 μL of 50 μM each primer, and 1 μL of the template.

The PCR products were purified and sequenced by SolGent (SolGent, Daejeon, Korea). The PCR conditions were initial denaturation at 94°C for 4 min, 30 amplification cycles of 94°C for 1 min, 56°C for 1 min, and 72°C for 2 min, and final elongation at 72°C for 10 min. Sequences were compared with published data in the GenBank databases (http://www.ncbi.nlm.nih.gov/genbank/). CLUSTAL-W in MEGA 6.06 software was used to align closely related sequences (Tamura et al., 2013). The neighbour–joining (NJ) method was employed to construct a phylogenetic tree for 16S with MEGA 6 after sequence alignment with Clustal W (version 7.222) (Katoh and Standley, 2013). Each node in the phylogenetic trees was statistically supported using 1000 bootstrap replications.

### Statistical analysis

Data are represented as the mean of three biological replicates ± the standard deviation (SD). The differences among groups were statistically analyzed via one-way analysis of variance (ANOVA) followed by a Duncan’s multiple range test (DMRT). The data were analyzed using IBM SPSS statistics v24.0 (SPSS Inc., Chicago, IL, USA). The criterion of statistical significance was set at the p value less than 0.05. A completely randomized design was adopted to compare the mean values of different treatments. A principal component analysis (PCA) and a Pearson correlation analysis were conducted to reveal the relationships within the selected parameters under the different conditions using the open-source statistical software R version 4.0. The functions corrplot and fviz_pca of R were used to generate a correlation–matrix plot and principal component analysis (PCA) biplot.

## Results

### Isolation of bacteria from different sources

In the present study, 81 bacterial strains were isolated from plants, rhizosphere, algae, lichen, sea sediments, and sea water from different locations in Muscat, Oman. **Table 1** shows the number of bacteria isolated from different sources.

### Characterization of bacterial isolates: IAA production, phosphate solubilization, and siderophore production

Various biochemical tests were performed to evaluate the growth-promoting effect of bacterial isolates; for initial screening, all these isolates were checked for IAA production by applying colorimetric assay using Salkowski reagents. Only 44 isolates from different sources exhibited positive results for IAA production (**Table 2**, **Supplementary Table 1** and **Supplementary Figure 1**). The highest IAA production was reported in GREB3 with 16.5 ± 0.26 μg/mL, followed by SPSB2 (15.38 ± 0.18 μg/mL) and GRRB3 (12.65 ± 0.13 μg/mL), and the lowest production of IAA was detected in TQEB1 with 0.13 ± 0.01 μg/mL (**Table 2** and **Supplementary Table 1**). The results presented in **Table 2** show the levels of soluble-P and the tricalcium phosphate (TCP) solubilization in NBRIP liquid medium by the tested strains. The concentration of soluble-P in the medium varied among different isolates, ranging from 0.18 μg/mL to 65.36 μg/mL. Among the strains tested, GREB3 exhibited the highest level of TCP solubilization in NBRIP liquid medium (65.36 ± 2.89 μg/mL), followed by SPSB2 (44.36 ± 3.24 μg/mL), GRRB3 (25.36 ± 0.04 μg/mL), and TQEF1 (20.42 ± 2.3 μg/mL). These results indicate that the tested strains have the ability to solubilize TCP in NBRIP liquid medium, with some strains displaying higher solubilization rates than others. The production of siderophores was assessed on solid CAS blue agar, and the formation of an orange halo around the colonies was observed, indicating the chelation of iron by the isolate in the medium. All isolates were tested for siderophore production, as shown in **Table 2** and **Supplementary Table 1**. Among the isolates, 21 exhibited high levels of siderophore production, while 4 demonstrated high performance. These findings suggest that the isolates have the ability to scavenge iron from their environment through the secretion of siderophores.

**Table 2:** Screening bacterial isolates for IAA, Siderophore production, and phosphate solubilization.

Isolates	IAA (ug/mL)	Phosphate Solubilization (ug/mL)	Siderophore	Isolates	IAA (ug/mL)	Phosphate Solubilization (ug/mL)	Siderophore
TQEB1	0.13 ± 0.01	20.42 ± 2.3	++	GRRB3	12.65 ± 0.13	25.36 ± 0.04	++
TQRB4	0.29 ± 0.02	17.12 ± 2.6	+	SPRB2	1.69 ± 0.10	30.65 ± 0.12	–
WSSB2	0.46 ± 0.01	11.36 ± 0.14	–	SPRB3	2.65 ± 0.08	2.36 ± 0.015	+
TQRB6	0.35 ± 0.03	1.45 ± 0.04	–	SPEB2	0.68 ± 0.02	1.96 ± 0.03	–
TQSB1	0.26 ± 0.01	1.89 ± 0.12	–	TQEB4	0.254 ± 0.03	0.36 ± 0.04	–
SDB1	12.9 ± 0.12	3.25 ± 0.02	+	SPEB4	8.98 ± 0.02	6.85 ± 0.18	+
SDB2	10.6 ± 0.10	4.87 ± 0.12	–	SPEB3	0.98 ± 0.06	–	–
TQEB2	0.65 ± 0.03	0.12 ± 0.03	–	SPSB2	15.38 ± 0.18	44.36 ± 3.24	+
SASB3	9.5 ± 0.06	3.98 ± 0.06	++	SPSB1	1.32 ± 0.01	–	–
MGRB2	0.98 ± 0.05	7.39 ± 0.036	–	WSEB3	7.96 ± 0.14	8.69 ± 0.07	+
MGRB4	0.25 ± 0.01	0.45 ± 0.04	–	WSRB4	5.69 ± 0.07	7.36 ± 0.069	+
MGEB2	0.48 ± 0.02	8.69 ± 0.85	–	LNB2	1.36 ± 0.03	4.63 ± 0.025	+
MGSB1	0.98 ± 0.06	12.36 ± 0.69	–	LNB4	0.65 ± 0.01	15.36 ± 0.89	+
GREB1	1.23 ± 0.08	0.18 ± 0.05	+	SAEB1	0.69 ± 0.03	13.98 ± 0.76	–
GRSB3	0.36 ± 0.01	0.89 ± 0.03	–	GREB4	0.98 ± 0.04	4.36 ± 0.15	–
GRRB1	8.65 ± 0.05	2.54 ± 0.12	+	GREB3	16.5 ± 0.26	65.36 ± 2.89	–

IAA and phosphate solubilization values are the mean of n = 3, expressed with standard error of means. -, Negative; +, Moderate; ++, High. The values are represented as mean ± standard deviation (SD). The mentioned isolates in this table exhibit at least two of the mentioned activities.

### Bioassay assessment of bacterial isolates on wheat seed germination

In order to evaluate their potential as plant growth-promoting agents, all isolates were subjected to a wheat seed germination bioassay. The findings indicated that nearly all of the isolated bacteria had an influence on seed germination (see **Table 3** and **Supplementary Table 2**). Among the 81 isolates tested, only eleven showed a significant enhancement in the average germination percentage of wheat compared to the control group. These eleven isolates comprised five endophytic, one rhizospheric, and five soil or sand isolates. The endophyte MGEB2, isolated from mangroves, profoundly affected wheat seed germination, increasing it by 66%. This effect persisted over time, with germination rates reaching 93% at 96 hours and ultimately reaching 100%. Several other isolates, such as TQEB3, GREB3, GRSB3, GRRB3, SPSB1, SPSB2, and SPEB4, significantly accelerated germination rates by up to 80-93% compared to the control group (which showed a germination rate of 53%) after four days. Other isolates, such as TQSB5 and TQSB7 (which increased germination rates by 60%) and SPEB2 (which increased germination rates by 63%), showed a slight improvement in germination rate compared to the control. Collectively, the findings from the study indicated that approximately 22% of the endophytic isolates and 14% of the sand or soil isolates exhibited a favorable influence on seed germination. Nevertheless, a significant proportion of these isolates displayed inhibitory effects on germination rates. Notably, isolates like LNEB1, SPEB3, SDB2, SDB3, 1WB1, and SPRB2 had a pronounced inhibitory impact, leading to the complete inhibition of wheat germination. This information is detailed in **Table 3** and **Supplementary Table 2**. The overall average germination percentage revealed that most rhizospheric isolates (94%) and water isolates (83%) had a detrimental effect on seed germination. These outcomes underscore the potential for utilizing certain isolated strains as agents that promote plant growth, while highlighting the possibility of negative impacts from others.

**Table 3:** Accumulated germination percentage of wheat seeds promoted by bacterial isolates over 6 Days.

Strains	Day 1	Day 2	Day 3	Day 4	Day 5	Day 6	Average
Control	0 ± 0	33.33 ± 0.57	40 ± 1.15	53.33 ± 0.57	66.66 ± 0.57	80 ± 1.15	45.55 ± 0.43*
TQEB2	26.66 ± 0.57*	40 ± 0*	60 ± 0*	60 ± 0*	60 ± 1*	60 ± 1*	51.11 ± 0.49*
TQEB3	33.33 ± 1.15*	53.33 ± 0.57*	66.66 ± 1.15*	66.66 ± 1.52*	80 ± 1*	80 ± 1	63.33 ± 0.30*
MGEB2	66.66 ± 1.52**	73.33 ± 1.52**	86.66 ± 2.08**	93.33 ± 1.15**	93.33 ± 0.57**	100 ± 0**	85.55 ± 0.74**
GREB3	73.33 ± 0.57***	73.33 ± 0.57**	80 ± 1**	80 ± 1*	80 ± 1*	80 ± 1	77.77 ± 0.22**
GRSB1	6.66 ± 0.57*	33.33 ± 0.57	53.33 ± 1*	60 ± 1*	73.33 ± 0.57*	73.3 ± 0.57*	49.99 ± 0.22*
GRSB3	6.66 ± 0.57*	26.66 ± 0.57*	46.66 ± 0.57*	80 ± 1*	86.66 ± 1.15**	86.6 ± 0.57*	55.55 ± 0.26*
GRRB3	46.66 ± 0.57*	60 ± 0**	73.33 ± 0.57**	73.33 ± 0.57*	73.33 ± 1.52*	73.33 ± 1.52*	66.66 ± 0.60**
SPEB4	33.33 ± 1.15*	53.33 ± 1.15*	73.33 ± 1.52**	73.33 ± 0.57*	80 ± 1*	86.66 ± 1.15*	66.66 ± 0.30**
SPSB1	6.66 ± 0.57*	26.66 ± 0.57*	46.66 ± 0.57*	60 ± 1*	73.33 ± 0.57*	86.66 ± 1.15*	49.99 ± 0.26*
SPSB2	40 ± 0**	53.33 ± 0.57*	80 ± 1**	80 ± 1*	80 ± 1*	93.33 ± 0.57*	71.11 ± 0.39**
SPSB3	13.33 ± 0.57*	26.66 ± 0*	53.33 ± 0.57*	60 ± 1*	60 ± 1*	66.66 ± 0.57*	46.66 ± 0.36*

The values are represented as mean ± standard deviation (SD). Stars represent statistical significance according to the one-way ANOVA test, p < 0.05*; p < 0.01**; p < 0.001***. The average shows the accumulative germination percentage after six days.

### Effect of bacterial isolates on wheat plant development and biomass

Similar to their positive impact on seed germination, these bacterial isolates also demonstrated beneficial effects on seedling growth parameters, including shoot and root length, as well as biomass, in comparison to control plants (as shown in **Table 4** and **Supplementary Table S**). Out of the 81 bacterial isolates examined for their influence on shoot growth, a subset of 15 isolates namely TQRB4, TQSB2, TQSB5, MGEB2, MGRB3, GREB2, GREB3, GRSB1, GRSB2, GRSB3, GRRB3, SPRB2, SPEB2, SPSB2, and SPSB3 demonstrated a significant increase in shoot length compared to the control (**Table 4** and **Supplementary Table 3**). Notably, it was observed that most rhizospheric isolates hindered wheat germination yet exhibited a positive effect on the length of wheat shoots. Specifically, approximately 33% of rhizospheric, 18% of endophytic, and 14% of sand or soil isolates were found to promote shoot length in wheat. Similar to the germination trend, around 22 bacterial isolates displayed inhibitory effects on shoot growth compared to the control (**Supplementary Table 3**). A similar pattern was observed in terms of root length, with most bacterial strains contributing to the promotion of wheat root growth. Among these, the most significant growth-promoting effect was observed in plants inoculated with GRSB1, followed by GRSB2, GRSB3, TQEB2, TQSB2, and TQSB1. Other isolates, including GRRB2, SPSB2, and SPSB3, also exhibited enhanced root length.

**Table 4:** List of bacterial isolates showing promotory effect on wheat seedling growth.

Strain	Shoot length	Root length	Fresh weight	Dry weight
Control	6.3 ± 0.57	2.13 ± 0.23	105.6 ± 14.04	46.0 ± 1.8
GREB2	8.03 ± 0.30*	2.56 ± 0.90	119.3 ± 8.14	38.4 ± 3.63
GREB3	9.23 ± 0.37**	3.36 ± 0.15*	168.6 ± 9.01*	76.7 ± 2.16*
GRRB3	10.13 ± 0.9**	4.16 ± 1.15*	175 ± 12*	72.9 ± 4.82*
GRSB1	8.56 ± 0.35*	7.06 ± 1.90**	166.6 ± 5.77*	47.5 ± 2.20
GRSB2	8.56 ± 1.40*	6.93 ± 0.51*	115.6 ± 0.57*	41.1 ± 1.40
GRSB3	8.46 ± 0.45*	6.13 ± 0.55*	130 ± 4.58*	37.8 ± 4.36*
MGEB2	8.26 ± 0.86*	2.8 ± 0.60*	124.3 ± 5.50*	57.3 ± 4.63*
MGRB3	8.9 ± 1.64*	3.1 ± 0.26*	164.3 ± 2.30*	54.2 ± 2.11*
SAEB2	6.83 ± 1.98	1.93 ± 0.37	118.9 ± 8.43*	56.9 ± 3.68
SPEB2	9.56 ± 1.05**	4.1 ± 1*	169.6 ± 8.32**	65.2 ± 4.02*
SPEB4	7.06 ± 0.60	1.26 ± 0.46	129.6 ± 9.07*	37.1 ± 5.71
SPRB2	8.63 ± 1.36*	2.93 ± 0.90*	158 ± 10.1*	75.5 ± 4.65*
SPRB3	8.4 ± 0.3*	2.66 ± 0.85	122 ± 12	65.5 ± 1.61*
SPSB2	7.73 ± 1.25	4.46 ± 0.85*	126.6 ± 3.51*	40.3 ± 5.5
SPSB3	9.43 ± 0.60**	3.3 ± 0.51*	186 ± 10.3*	75.2 ± 1.90**
TQEB2	8 ± 1.32	4.1 ± 1.27*	168 ± 10.5*	48.7 ± 2.85*
TQEB3	7.26 ± 0.87	2.33 ± 0.56	121 ± 5.56	38.4 ± 2.75
TQRB4	9.63 ± 1.76**	3.6 ± 0.36*	161.3 ± 16.2*	39.9 ± 2.34
TQRB5	8.16 ± 0.70	2.2 ± 0.51	158.3 ± 16.1*	39.8 ± 6.21
TQRB6	8.96 ± 0.45*	2.9 ± 0.95	119 ± 16.5	50.4 ± 7.66
TQSB2	8.16 ± 2.36	5.6 ± 1.44*	122.3 ± 24.0*	42.1 ± 5.42
TQSB5	8.96 ± 1.12*	3.93 ± 0.30*	172.3 ± 4.04**	84.3 ± 5.32***

The values are represented as mean ± standard deviation (SD). Stars represent statistical significance according to the one-way ANOVA test, p < 0.05*; p < 0.01**; p < 0.001***.

On the contrary, certain isolates like 2WB1, SDB1, SDB3, SDEB1, TQRB7, TQRB2, and TQRB3 displayed a notable inhibitory effect on root growth (**Supplementary Table 3**). In the context of both shoot and root length, it is noteworthy that 44% of the rhizospheric isolates exhibited an inhibitory effect, whereas 22% displayed a growth-promoting effect on shoot and root length, respectively. Fresh weight results also revealed that TQSB1 and TQSB2 significantly increased fresh weight, followed by GRRB3 and SPSB3, while the MGRB1 and MGRB2 ultimately reduced it as compared to the control (**Table 4** and **Supplementary Table 3**). Among the tested isolates, GREB3, GRRB3, and SPSB3 were found to enhance all growth parameters as compared to the control significantly. Based on these findings, these three isolates were selected for further investigation to determine their potential to mitigate the detrimental effects of salt stress on plant growth. The selection was based on the observation that these isolates consistently demonstrated the highest level of growth promotion under non-saline conditions, suggesting that they may have a greater potential to alleviate the negative impact of salt stress on plant growth. The next phase of the study will aim to identify these isolates and evaluate the effectiveness of these isolates in promoting growth and mitigating the adverse effects of salt stress on wheat plants.

### Principal component analysis (PCA) and wheat germination

A principal component analysis (PCA) was performed on 81 bacterial cultural filtrates obtained from diverse sources, including plants, algae, seawater, lichen, and sea sands, for biopriming wheat seeds and examining the germination percentage over a period of six days (**Supplementary Figure 2**; **Supplementary Table 4**). The results of the PCA revealed that the first two principal components (PCs), which exhibited the highest variance (PC1: 86.2%; PC2: 10%), accounted for 96.16% of the total variation. A PCA-biplot was then constructed using only these two PCs. The analysis revealed that the bacteria isolated from various sources had varying effects on the germination percentage of wheat, as evidenced by the grouping of treatments in the PCA plot. Notably, wheat seeds treated with bacteria isolated from algae, *Paspalum vagintum*, and *Tetraena qatarensis* were separated from the control treatment during the first five days. Additionally, significant separation was observed between rhizospheric and endophytic bacteria treatments compared to the control. Overall, this study provides valuable insights into the effects of different bacterial sources on wheat seed germination, which can have important implications for biopriming strategies in agricultural systems.

Continuing with the investigation of the effects of bacterial strains on wheat growth, a principal component analysis (PCA) was conducted on the same set of 81 bacterial strains using wheat seedling growth parameters, including shoot length (SL), root length (RL), fresh weight (FW), and dry weight (DW). The results of the PCA revealed that the first two principal components (PCs) with eigenvalues >1 accounted for 86.16% of the total variation (**Supplementary Table 5**), with PC1 contributing the majority of the variance (66.3%) and PC2 contributing 7%. Based on these findings, a PCA-biplot was constructed using only PC1 and PC2 (**Supplementary Figure 3A**). The PCA analysis showed that the bacteria isolated from different sources had varying effects on the wheat growth parameters, as demonstrated by the grouping of treatments in the PCA plot (**Supplementary Figure 3B**). Notably, the wheat seeds treated with mostly rhizospheric, endophytic, and algae-derived bacteria were separated from the control treatment in terms of RL, SL, FW, and DW. Furthermore, as with the germination percentage, the bacterial strains isolated from different plants exhibited distinct effects on wheat growth parameters. Bacteria isolated from algae, *Tetraena qatarensis*, and *Paspalum vaginatum* were particularly noteworthy, as they demonstrated significant separation from the control treatment in terms of all four growth parameters (**Supplementary Figure 3B**).

### Bacterial identification and phylogenetic analysis

**Figure 1:**
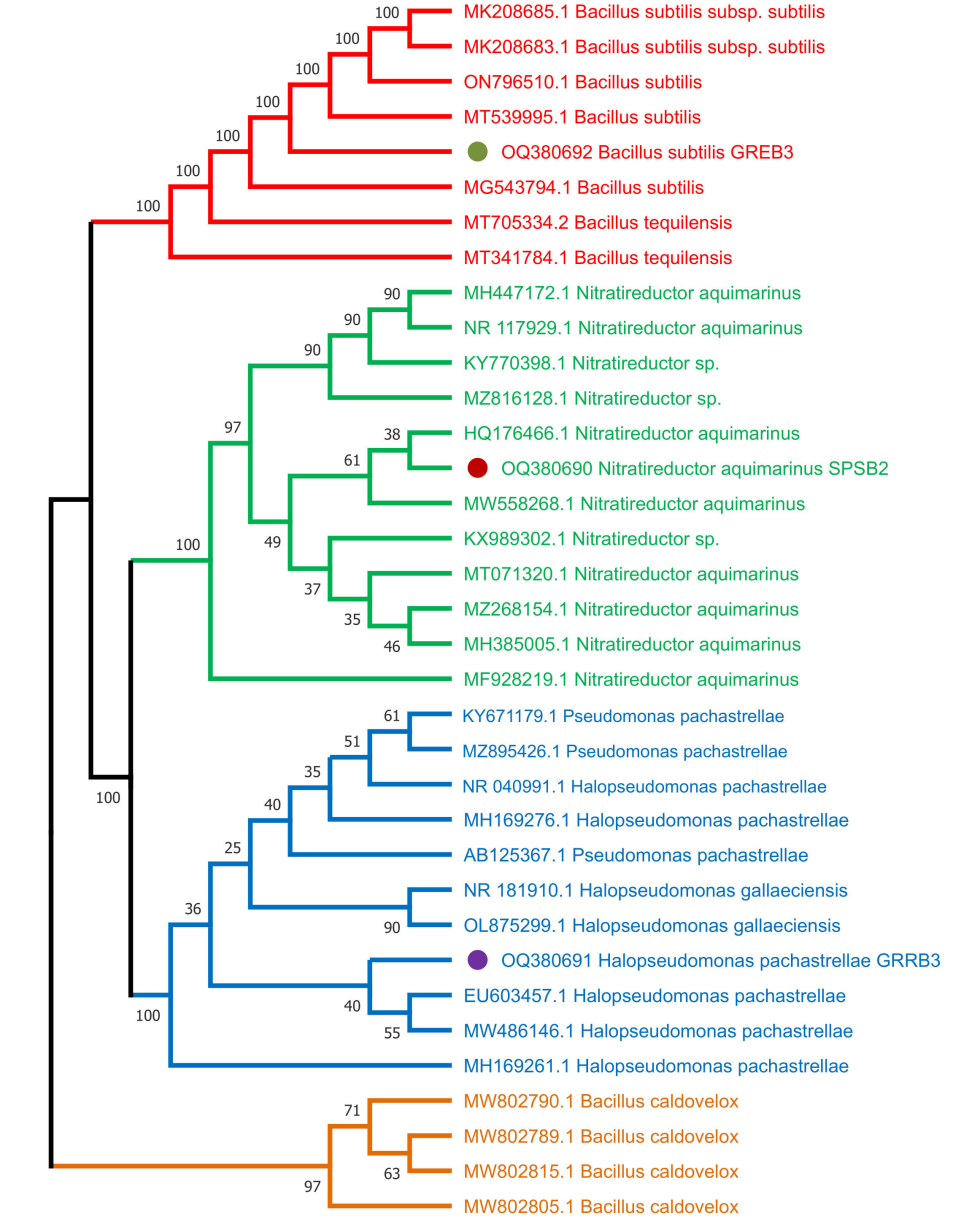
Molecular phylogenetic analysis of three bacterial strains used in this study from 16S region using neighbour joining (NJ) method.

To identify the selected three bacterial strains SPSB2 (OQ380690), GRRB3 (OQ380691), and GREB3 (OQ380692), and to infer their phylogenetic position, the sequenced 16S ribosomal RNA, the isolates have been compared to the sequences in the NCBI database through BLAST search analysis (http://www.ncbi.nlm.nih.gov/). The results revealed that the SPSB2 exhibited a higher level of 16S sequence identity to *Nitratireductor aquimarinus*, GRRB3 showed similarity with *Halospseudomonas pachastrellae*, and GREB3 showed similarity with Bacillus subtilis respectively. The neighbour–joining (NJ) method was employed to construct a phylogenetic tree for 16S with MEGA 6 after sequence alignment with Clustal W (version 7.222) (Katoh and Standley, 2013), keeping default parameters. The results revealed that based on 16S regions, SPSB2 formed a single clade with *Nitratireductor aquimarinus*, while GRRB3 grouped with *Halospseudomonas pachastrellae*, GREB3 formed a clade with Bacillus subtilis supported by a relatively strong bootstrap value (**Figure 1**).

### Effect of bacterial isolates on wheat seedlings under salt stress

**Figure 2:**
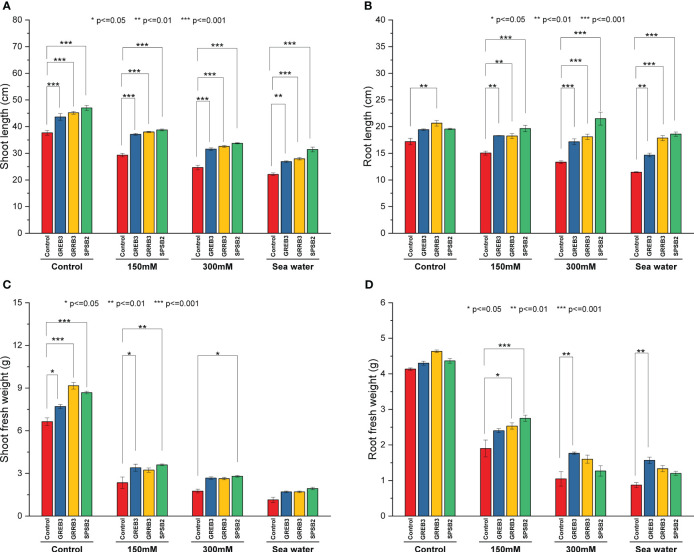
Effect of growth-promoting bacteria on wheat growth parameters under 150 mM, 300 mM NaCl, and seawater (100%) stress. **(A)** Shoot length, **(B)** Root length, **(C)** Shoot fresh weight, and **(D)** Root fresh weight. The data shown are the means of three biological replicates (± SD). Statistical testing were carried out using one-way ANOVA, and asterisks above plots indicate significant differences between inoculated and non-inoculated plants. Significant differences are indicated (ns, not significant; *P = 0.05, **P = 0.01, ***P = 0.001).

The present study investigated the plant growth promotion potential of bacterial isolates, which were previously identified to possess such properties GREB3, GRRB3, and SPSB2. Specifically, we evaluated their efficacy in promoting the growth of wheat plants under salt-stress conditions. Our findings revealed that salt stress significantly inhibited wheat plant growth, with a positive correlation between the degree of inhibition and increasing salt concentration. In particular, seawater was found to exert a strong inhibitory effect on shoot growth compared to the control (**Figure 2** and **Supplementary Figure 4**). A reduction of 22%, 34%, and 41% was observed at 150 mM, 300 mM, and SW salt stress, respectively, as compared to control plants. The GREB3 increased shoot length up to 15%, GRRB3 up to 16%, and SPSB2 up to 24% as compared to the control. In combination with salt stress, the bacterial isolates SPSB2 significantly alleviate the effect of salt stress and promote plant shoot length as compared to their respective salt stress concentrations (150 mM, 300 mM, and SW) (**Figure 2A**). Like shoot growth, the root growth was also inhibited by salt concentrations, reducing root length up to 12% at 150 mM, 22% at 300 mM, and 31.2% at SW-treated plants. All the selected isolates exhibited a more promotory effect on root length, but GRRB3 significantly increased root length and alleviated the effect of salt stress at 150, 6%, 300 mM 5.2%, and seawater 3.7% as compared to control (**Figure 2B**). A similar increase was noted in the case of plants inoculated with SPSB2, where it increases the root growth and mitigates the effect of salt stress up to 14%, 25%, and 8% at 150 mM, and 300 mM seawater, respectively. Like root and shoot length, the fresh weight was negatively affected by salt stress at 150 mM 64%, at 300 mM 73%, and at seawater, 82% reduction was reported. However, the plant inoculated with bacterial isolates showed a significantly increased shoot fresh weight compared to the control (**Figure 2C**). Like other growth parameters, the fresh root biomass was reduced by NaCl concentration compared to the control (**Figure 2D**).

### Determination of flavonols, flavonoids, and total proteins

**Figure 3:**
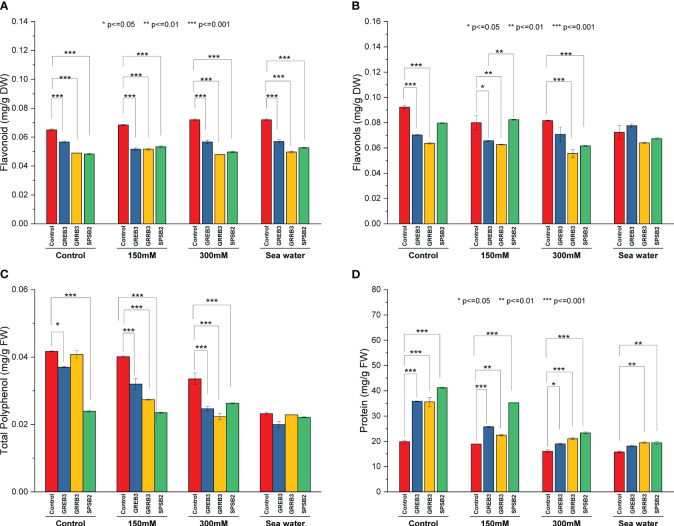
Effect of growth-promoting bacteria on wheat growth parameters under 150 mM, 300 mM NaCl and seawater (100%) stress. **(A)** Flavonoid, **(B)** Flavenol, **(C)** Total Polyphenol, and **(D)** Total protein. The data shown are the means of three biological replicates (± SD). Statistical testing were carried out using one-way ANOVA, and asterisks above plots indicate significant differences between inoculated and non-inoculated plants. Significant differences are indicated (ns, not significant; *P = 0.05, **P = 0.01, ***P = 0.001).

Salt stress and bacterial inoculation also affected secondary plant metabolites like flavonols and flavonoids. The results showed that the bacterial isolates decreased the concentration of flavonols and flavonoids compared to control and simple salt treatments. However, the salt dilutions significantly promote its concentration. All three isolates significantly reduce flavonols contents compared to control plants in flavonols. The GREB3 showed a similar result, and no significant change was observed in flavonols contents when applied in combination with seawater compared to normal seawater (**Figure 3A**). Like flavonols, the flavonoid content is also negatively affected by the bacterial isolates in wheat plants (**Figure 3B**). The GRRB3 and SPSB2 significantly decreased the flavonoid content in plants. The protein content result showed that control and salt dilution significantly reduced the protein content of the wheat plants. All three species significantly increase the protein content. The isolates GREB3 and SPSB2 significantly increase the protein content at 150 mM concentration as compared to the control (**Figure 3D**).

### Determination of catalase, polyphenol oxidase activity

**Figure 4:**
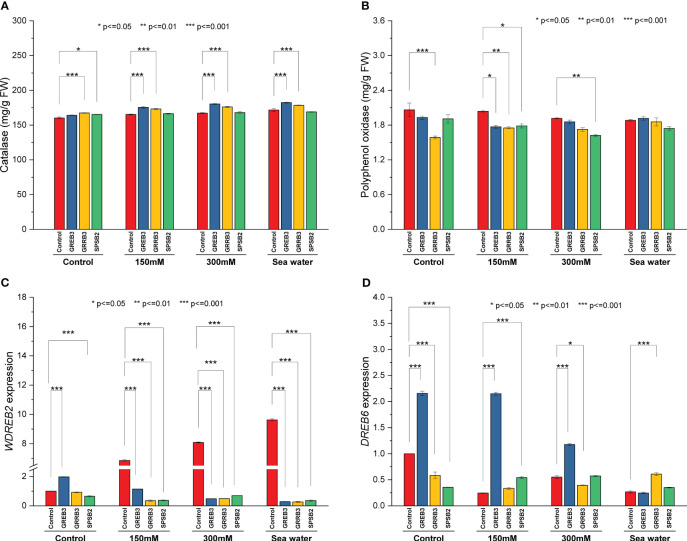
Effect of growth-promoting bacteria on wheat growth parameters under 150 mM, 300 mM NaCl and seawater (100%) stress. **(A)** Catalase, **(B)** polyphenol oxidase, **(C)** *WDREB2* gene expression, and **(D)** *DREB6* gene expression. The data shown are the means of three biological replicates (± SD). Statistical testing were carried out using one-way ANOVA, and asterisks above plots indicate significant differences between inoculated and non-inoculated plants. Significant differences are indicated (ns, not significant; *P = 0.05, **P = 0.01, ***P = 0.001).

The catalase and polyphenol oxidase activities were determined in plants inoculated with bacterial isolates under salt stress. The result shows that salt stress decreased the catalase activity compared to the control (**Figure 4A**). The isolate GREB3 slightly increased the catalase activity with seawater (0.7%). The GRREB3 at 150 mM showed a 0.5% increase in catalase activity compared to the control, and the isolate in combination with SPSB2 + 300 mM showed a 0.4% increase. The polyphenol oxidase activity also decreased in bacterial-treated plants as compared to control and simple salt concentrations. In the case of GREB3, the effect of GREB3 + 150 mM is more prominent, while in the case of SPSB2, the effect of SPSB2 + 300 mM is more inhibitory (**Figure 4B**). The result of total polyphenol content showed that the bacterial isolate GREB3, combined with salt stress, increases the plant’s polyphenol content upt 23% at 150 mM, 40% at 300 mM and 52% in seawater treated plantsThe GRRB3 also increased the total polyphenol in combination with 300 mM salt dilution (**Figure 3C**).

### Gene expression under salt stress and bacterial inoculation

The relative expression of genes associated with abiotic stress were examined to determine the molecular mechanism behind the reduction of salt stress in wheat plants. In the present study, we employed qPCR to assess the expression levels of *DREB6* (AY781361.1) and *WDREB2* (AB193608.1) genes. These genes have been previously documented to exhibit a close association with osmotic stress conditions, including drought and salt stress. Notably, they play a pivotal role in regulating promoter methylation, a crucial mechanism involved in modulating DNA methylation patterns during a plant’s response to osmotic stress. However, the *WDREB2* gene was found to be highly upregulated during salt stress conditions (**Figure 4C**). This upregulation was directly proportional to salt concentration. However, the expression level of *DREB6* was downregulated under salinity stress (**Figure 4D**). In the case of inoculated plants, all three bacterial isolates downregulated the *WDREB2* gene expression compared to respective salt stress-treated plants. However, GREB3 exhibited upregulation under normal control conditions but downregulation under salt stress conditions. On the contrary, DREB6 expression was found to be upregulated after inoculation with GREB3 in control and in both 150 mM and 300 mM stressed plants. However, GRRB3 and SPSB2 isolated did not show any significant effect, and similar downregulation was observed as compared to control plants.

### Principal component analysis and correlation of traits

**Figure 5:**
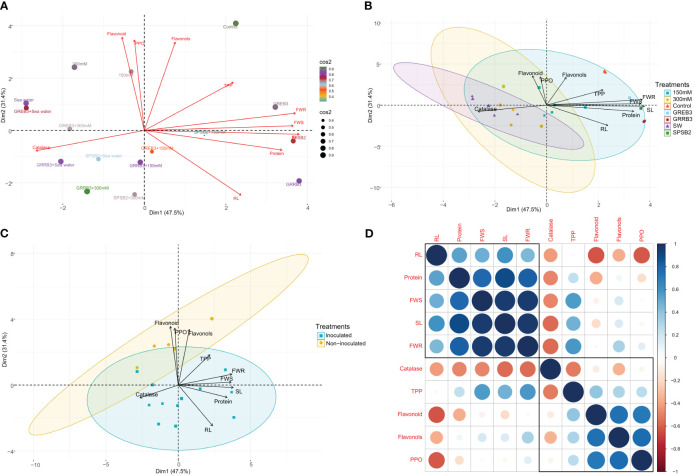
**(A)** Principal component analysis (PCA) biplot of individual wheat plant based on the variance in morpho-physiological, biochemical, and antioxidative traits under control, salt stress, inoculated, and un-inoculated conditions. **(B)** Principal component analysis (PCA) biplot of wheat plant based on the variance in morpho-physiological, biochemical, and antioxidative traits under control, salt treated, and bacterial inoculated treatments. The length of the arrows’ length indicates the attributes’ contribution to the first two components of PCA. **(C)** Principal component analysis (PCA) biplot of wheat plant based on the variance in morpho-physiological, biochemical, and antioxidative traits during inoculated and non-inoculated plants. **(D)** Pearson’s correlation matrix between plant growth attributes, antioxidant enzymes, and secondary metabolites in salt-stressed inoculated and non-inoculated plants. Correlations are displayed in blue (positive) and red (negative); color intensity and circle size are proportional to the correlation coefficient. RL, root length; SL, shoot length; FWS, shoot fresh weight; FWR, root fresh weight, Protein, Catalase, Flavonoid, Flavonols; TPP, total polyphenol and PPO, polyphenol oxidase.

This study investigated the variability in morpho-physiological, biochemical, and antioxidative traits of individual wheat plants under control, salt stress, and bacterial inoculated and un-inoculated conditions using principal component analysis (PCA). The results revealed that the first principal component (PC1) accounted for 47.5% of the total variability among traits and was mainly associated with shoot length (SL), root length (RL), and protein. In comparison, the second principal component (PC2) accounted for an additional 31.4% of the variability and appeared to be related to root and shoot fresh weight (FWR, FWS), flavonols, and total polyphenol (TPP) in response to salt stress inoculated and non-inoculated treatments (**Figure 5A**). Moreover, the PCA analysis showed that most of the bacterial treatments in the control conditions were well represented in PC1 and PC2, and they had a significant effect on SL, RL, FWR, FWS, flavonols, and proteins (**Figure 5B**). Additionally, we observed that most of the traits were positively correlated in both inoculated and non-inoculated treatments and presented in PC1 and PC2, while catalase, flavonoids, flavonols, and PPO were negatively correlated with plant morphological traits and related to non-inoculated treatments (**Figures 5C, D**).

Furthermore, we performed a Pearson correlation analysis to investigate the extent of the relationship among the traits. The results showed significant correlations among the morphological, biochemical, and antioxidative traits under salt stress and control conditions in both inoculated and non-inoculated plants. Interestingly, the protein was found to be positively correlated with other morphological traits such as SL, RL, FWS, and FWR. On the other hand, Catalase, flavonoids, flavonols, and PPO were negatively associated with plant morphological traits. Furthermore, TPP and flavonols were found to be negatively correlated with RL, while positively correlated with SL (**Figure 5D**). Overall, these findings provide insights into the complex interactions among different traits in wheat plants under different conditions and highlight the potential of using bacterial inoculants to improve plant growth and stress tolerance.

## Discussion

The significant climate change affecting today’s agriculture sector and the resulting salt intrusion have reduced coastal agricultural fields, leading to food insecurity and unsustainability for the growing population worldwide (Nabti et al., 2015; Shrivastava and Kumar, 2015; Szabo et al., 2016; Ansari et al., 2019). Different irrigation techniques, conventional breeding, and genetic engineering of salt-tolerant transgenic plants are currently used, but these procedures are very labor and technically intensive, making them difficult to be implemented (Singh et al., 2015; Niu et al., 2018). Climate-smart agriculture now includes the application of PGPR in the form of bioinoculants and biofertilizers to combat salt stress and increase crop yields in salinity-prone coastal agricultural sites. We initially screened bacteria under normal conditions, rather than abiotic stress, to screen out whether they exhibit plant growth promotion under optimal conditions. The results show that different bacterial isolates exhibited a promotory effect on wheat plant growth; these results were identical to those (Huang et al., 2015; Takishita et al., 2018) that seed treatment and root inoculation with particular bacterial isolates resulted in consistently positive performance in Petri dish experiments. The findings from the study revealed exciting insights into the effects of bacteria from different sources on seed germination and plant growth. Notably, a higher percentage of endophytic isolates (22.7%) exhibited a promotory effect compared to rhizospheric (5.5%) and soil (14.2%) isolates.

Conversely, a significant portion of rhizospheric isolates (94%) demonstrated an inhibitory effect, followed by soil and water isolates, as seen in both **Table 3** and **Supplementary Table 2**. Of particular interest was the observation that a substantial majority of water isolates (83%) exhibited inhibitory effects on seed germination. These outcomes underline the varying impact of isolates from diverse ecological niches on seed germination, with a notable prevalence of promotory effects among endophytic isolates. However, the analysis of other plant parameters, such as root length, shoot length, and fresh and dry weight, did not exhibit significant differences concerning the bacterial source. Roughly 18% of endophytic isolates demonstrated promotory effects on both root and shoot length, while 33% and 22% of rhizospheric isolates displayed promotory effects on shoot and root length, respectively. Surprisingly, none of the water isolates were found to have a promotory effect on any wheat growth parameters. While seed germination outcomes displayed variability based on the source of isolated bacteria, the overall growth of wheat plants suggested that the bacterial source might not be a decisive factor, except for water isolates, which hindered various plant growth parameters. Earlier research on endophytic and rhizospheric bacteria similarly concluded that the original ecological niches might not be the primary determinant of growth-promoting attributes (Wang et al., 2022; Wen et al., 2023). Previous reports have indicated that PGPR foster plant growth through diverse mechanisms, including hormone production such as IAA, ABA, GA, and cytokinins (Patten and Glick, 2002; Dobbelaere et al., 2003), symbiotic nitrogen fixation (Kennedy et al., 1997; Kennedy et al., 2004), and nutrient solubilization (Richardson, 2001; Hayat et al., 2010; Kang et al., 2017).

These results are consistent with earlier work that IAA is the most significant phytohormone, and bacteria with IAA-producing capacity can promote plant root growth and length, producing a greater root surface area that helps the plant to absorb more nutrients from the soil (Islam et al., 2016). The shoot length and fresh biomass were also significantly increased by plants inoculated with bacterial isolates, and our results agree with previous reports that IAA production by endophytic bacterial species is beneficial for promoting plant growth (Karnwal, 2009). Conversely, several studies have noted that microbial metabolites can contain natural compounds that hinder seed germination (Abo-Elyousr et al., 2021; He et al., 2022). For instance, Zonno and Vurro (2002) found that certain toxins produced by bacteria and fungi hindered the germination of *Orobanche ramosa* seeds, suggesting potential use in controlling parasitic plants. Similarly, Abo-Elyousr et al. (2021) demonstrated that *P. putida* ASU15 inhibited *U. appendiculatus* urediniospore germination by increasing bacterial concentration. In our research, we observed inhibitory effects on wheat seed germination and seedling vigor from diverse endophytic and rhizospheric isolates from different sources. This observation corresponds with Xiao et al. (2020), reporting that rhizobacteria, specifically *Bacillus* spp. isolated from bean plants, decreased rice seed germination. These isolates might hold promise as natural herbicides for weed seed germination management in the future. Nevertheless, their soil stability and potential toxicological implications warrant further investigation.

Salinity negatively affects plant growth and yield, directly impacting the soil’s biological and physiochemical properties. Specific ion toxicity, osmotic stress, nutritional disruption, or combinations of these elements are responsible for salinity’s detrimental effects on plant development (Parida and Das, 2005; Ju et al., 2021). In the present study, plant growth was inhibited by both seawater and NaCl, and this inhibition increased with concentration. However, the bacterial strains alleviated the stress caused by NaCl on wheat plants. Previously, similar result was described in *G. max* inoculated with bacterial strain SAK1 by (Khan et al., 2020). In early Studies numerous plant species have been treated with endophytic bacteria to reduce salt stress (Pal et al., 2021; Sofy et al., 2021). Similarly, the inoculation of *E. ludwigii* B30 improved plant growth in both non-salt stress and salt stress environments, as evidenced by higher shoot height, root length, and shoot and root biomass (Wei et al., 2022). The antioxidative enzymes produced by PGPB or the expression of plant genes involved in the synthesis of ROS-scavengers can both help plants recover from oxidative stress. PGPB can alleviate oxidative stress in plants by producing antioxidative enzymes or regulating the expression of plant genes involved in synthesizing ROS scavengers (Mishra et al., 2021). In our study, the bacterial isolates increased the catalase activity as compared to plants treated with NaCl, while the polyphenol oxidase activity was reduced. Similar to our result it was reported in early studies that in marginalized agricultural systems, the rhizobacteria *Bacillus licheniformis* and *Pseudomonas plecoglossicida* support the growth of the sunflower plant under salt stress by promoting the expression of the antioxidant enzymes CAT, SOD, and GPX as well as the production of ACC deaminase (ACCD) and IAA (Yasmeen et al., 2020). Some non-enzymatic antioxidants produced by PGPB, in addition to the antioxidative enzymes, play a substantial role in reducing the effects of oxidative stress in plants exposed to salinity.

In present study the protein and flavonole contents of plants increased in bacterial inoculated plants separately or in combination with a NaCl stress as compared to simple salt treatment while flavonoid content showed similar result in salt as well as bacteria + NaCl. The result shows resemblance with other studies like the medicinal herb *Artemisia annua* L. has been shown to grow and produce more effectively when *Piriformospora indica* and *Azotobacter chroococcum* are inoculated to plants. These PGPB reduced the oxidative damage in plants by lowering the amount of (malondialdehyde) MDA and increasing the amount of enzymatic as well as non-enzymatic antioxidants such phenolics, flavonoids, and carotenoids components (Arora et al., 2020; Kumar Arora et al., 2020). In a similar vein, the work of (Batool et al., 2020) demonstrated that treating *Solanum tuberosum* with *Bacillus subtilis* led to an elevation in free amino acid content. Proline, a significant amino acid, serves multiple functions in countering various abiotic stresses, acting as an osmoprotectant, cell structure stabilizer, and scavenger of reactive oxygen species (ROS) (Abbasi et al., 2020). Moreover, the research by Prittesh et al. (2020) indicated that a range of bacteria, including *Bacillus* sp., *Exiguobacterium* sp., *Enterobacter* sp., *Lysinibacillus* sp., *Stenotrophomonas* sp., *Microbacterium* sp., and *Achromobacter* sp., can heighten proline levels in rice.

The expression of genes related to salt tolerance and genes encoding antioxidant enzymes was dramatically upregulated by PGPR inoculation (Ali et al., 2022a). The first transcription factors associated with regulating gene expression in response to abiotic stresses were the DREB genes. The overexpression of *DREB2A* under conditions of high salinity and drought stress reveals that the DREB protein is essential for expressing genes that respond to dehydration (Moran et al., 1994; Tiwari et al., 2017). The present study reported the expression of two osmotic stress-related genes, *DREB6* (AY781361.1) and *WDREB2* (AB193608.1). In selected isolates, the GREB3 upregulated the expression of these genes as compared to control and salt stress. This result is consistent with previous work on sugarcane plants in which the *DREB2A* is overexpressed due to the use of PGPR and SNP (sodium nitroprusside), which may help the host plant quickly withstand stress (Sharma et al., 2021). The findings of this investigation are also consistent with those of Augustine et al. (Augustine et al., 2015), who reported that in sugarcane, salinity and drought stress were augmented by the overexpression of the *Erianthus arundinaceou*s *DREB2* gene.

Our study has shown that bacteria collected from different sources, whether they are endophytic (GREB3), rhizospheric (GRRB3), or from soil/sand (SPSB2), can exhibit similar promotory effects in mitigating salt stress conditions in plants. On the other hand, we’ve also found that bacteria from the same source and with similar characteristics (endophytic or rhizospheric) can lead to varying outcomes in terms of germination and plant growth attributes. This suggests a complex relationship between bacteria and plant responses that goes beyond their origin or nature.

## Conclusion

In conclusion, the present study highlights the effects of salt stress and bacterial inoculation on various morphological, physiological, biochemical, and antioxidative parameters in wheat plants. The results indicate that salt stress negatively impacts plant growth, while bacterial inoculation can mitigate the harmful effects of salt stress to some extent. The bacterial isolates GREB3, GRRB3, and SPSB2 showed different effects on plant growth and antioxidative parameters under salt stress conditions. The study also revealed that salt stress and bacterial inoculation significantly affected the concentration of flavonols, flavonoids, and total proteins. Flavonols and flavonoids were found to decrease under bacterial inoculation while the protein content increased. The catalase and polyphenol oxidase activities were also significantly affected by salt stress and bacterial inoculation, indicating the potential role of bacterial inoculation in regulating plant stress responses. Moreover, gene expression analysis revealed the involvement of specific genes such as *WDREB*2 and *DREB*6 in plant stress responses, and their expression was differentially regulated by salt stress and bacterial inoculation. Principal component analysis and Pearson correlation analysis further supported the findings of the study and demonstrated the significant relationships among various parameters under different stress conditions. Overall, the study provides valuable insights into the mechanisms underlying plant stress responses and the potential use of bacterial inoculation as a sustainable approach to mitigate the harmful effects of salt stress in crops.

## Data availability statement

The original contributions presented in the study are included in the article/**Supplementary Material**. Further inquiries can be directed to the corresponding authors.

## Author contributions

MA, L, WA, and SAsaf performed experimental and analysis. RJ, SAsif, and AK extracted DNA and microbes identification; SB performed phytohormones and antioxidant analysis; SAsaf, MW, and IK wrote the draft manuscript and statistical analysis. K-MK and AA-H, supervision and arranging resources. All authors contributed to the article and approved the submitted version.

## Supplementary material

Supplementary Figure 1 Variable color responses depending on the IAA amount using Salkowski’s reagent biosynthesized by isolated strains.

Supplementary Figure 2
**(A)** Principal component analysis (PCA) showing bioassay assessment of bacterial isolates (collected from different sources) on wheat seed germination percentage. Day 1, Day 2, Day 3, Day 4, Day 5 and Day 6 indicate germination percentage after 24h, 48h, 72h, 96h, and 120h. **(A)** Different shapes indicate the source from where bacteria were isolated, and **(B)** Different shapes indicate the nature or source of bacteria source from where bacteria were isolated.

Supplementary Figure 3 Principal component analysis (PCA) shows bioassay assessment of bacterial isolates (collected from different sources) on wheat seedling growth parameters. SL, RL, FW, and DW indicate shoot length, root length, and fresh, and dry weight of wheat seedlings. **(A)** Different shapes indicate the source from where bacteria were isolated, and **(B)** Different shapes indicate the nature or source of bacteria source from where bacteria were isolated.

Supplementary Figure 4 Effect of growth-promoting bacteria on wheat growth parameters under 150 mM, 300 mM NaCl, and seawater (100%) stress. **(A)** Effect of salt stress on wheat plant growth, **(B)** Effect of growth promoting bacteria on plant growth under normal conditions, **(C)** Effect of growth promoting bacteria on wheat growth under 150 mM NaCl stress, **(D)** Effect of growth promoting bacteria on wheat growth under 300 mM NaCl stress and **(E)** Effect of growth promoting bacteria on wheat growth under seawater stress.
