# Supplementary material for: Exploring the potential of halotolerant bacteria from coastal regions to mitigate salinity stress in wheat: physiological, molecular, and biochemical insights
Source: Front Plant Sci. 2023 Sep 22;14:1224731. doi: 10.3389/fpls.2023.1224731 (PMC10556533; doi:10.3389/fpls.2023.1224731)
Supplement: Supplementary Figure 1 — Variable color responses depending on the IAA amount using Salkowski’s reagent biosynthesized by isolated strains. [file DataSheet_1.pdf]

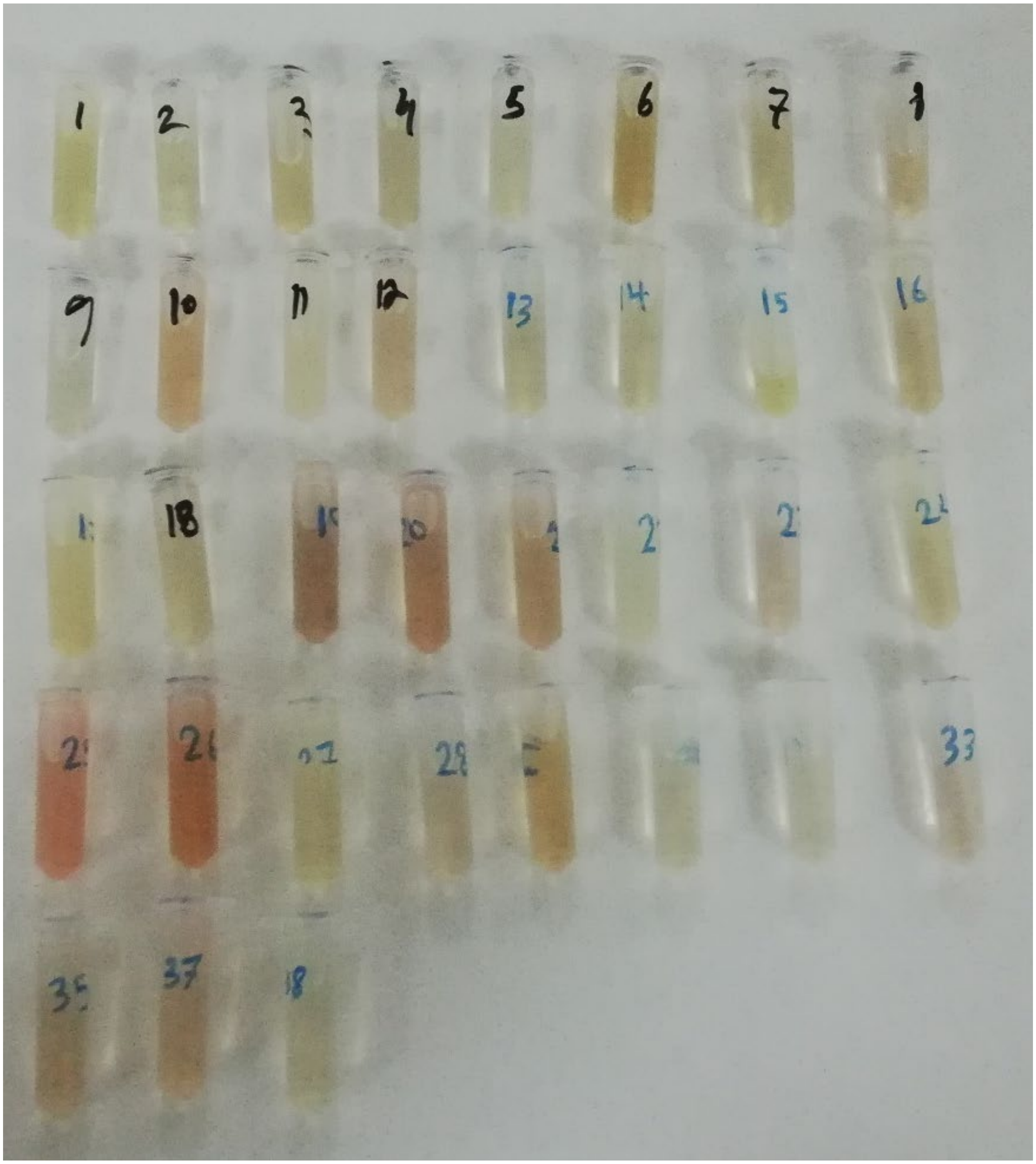
1
2
3
4
5
6
7
9
10
11
12
13
14
15
16
18
20
33
37

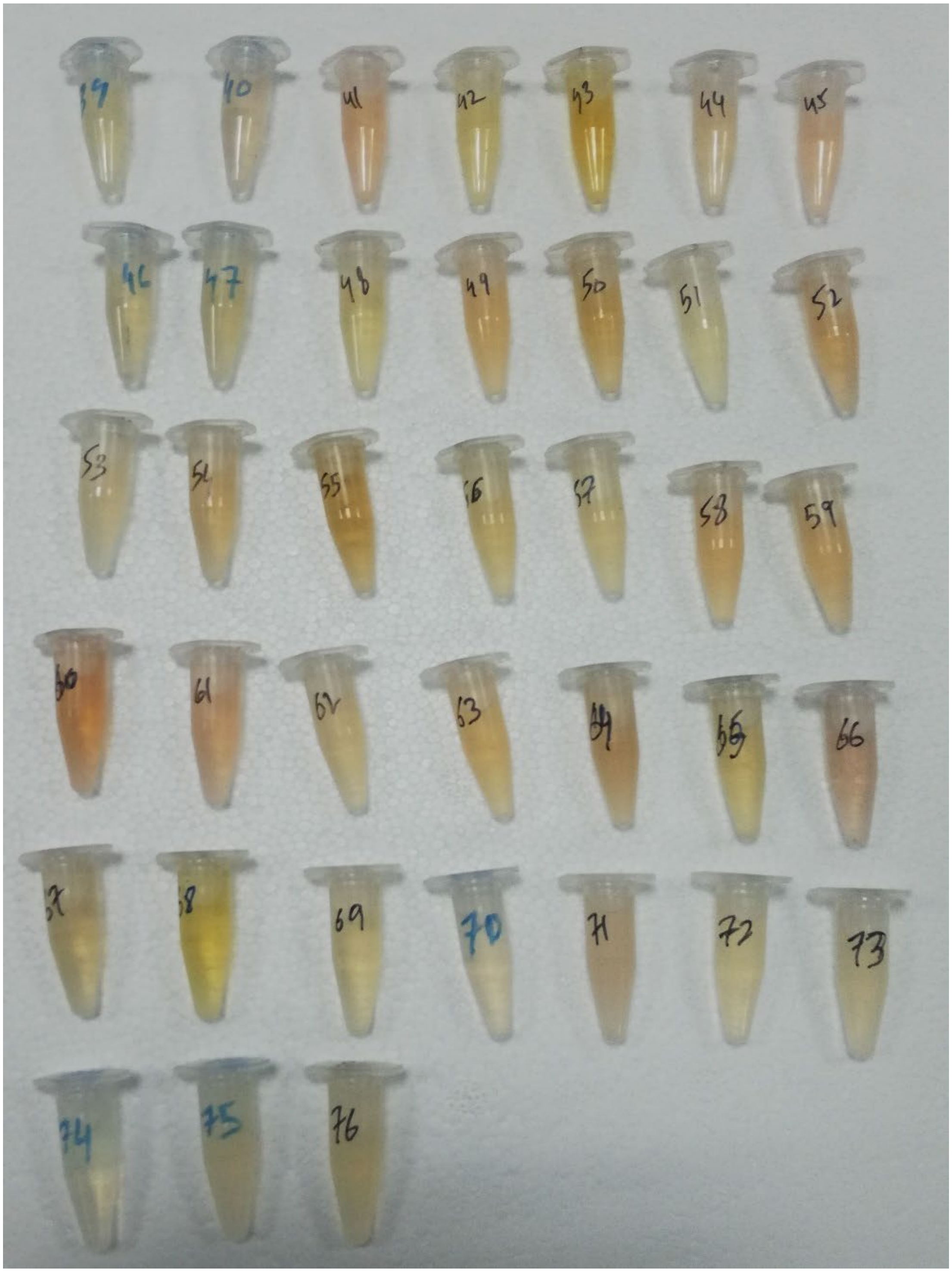
40
41
42
43
44
45
46
47
48
49
50
51
52
53
54
55
56
57
58
59
60
61
62
63
66
69
70
71
72
73
74
75
76

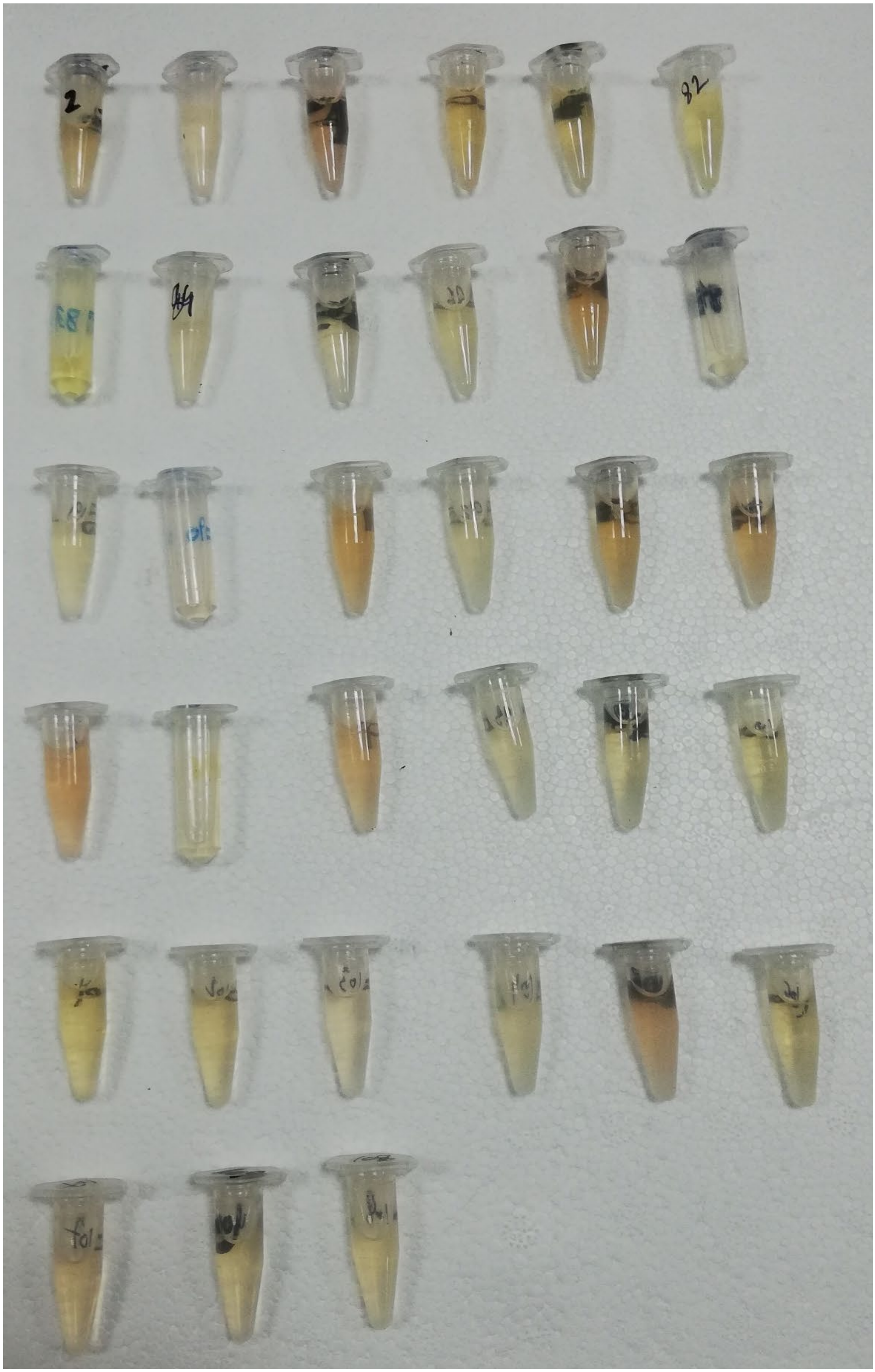
2
82

Figure S1. Variable color responses depending on the IAA amount using Salkowski's reagent biosynthesized by isolated strains.
