# Supplementary figures and images for: Exploring the potential of halotolerant bacteria from coastal regions to mitigate salinity stress in wheat: physiological, molecular, and biochemical insights
Source: Front Plant Sci. 2023 Sep 22;14:1224731. doi: 10.3389/fpls.2023.1224731 (PMC10556533; doi:10.3389/fpls.2023.1224731)

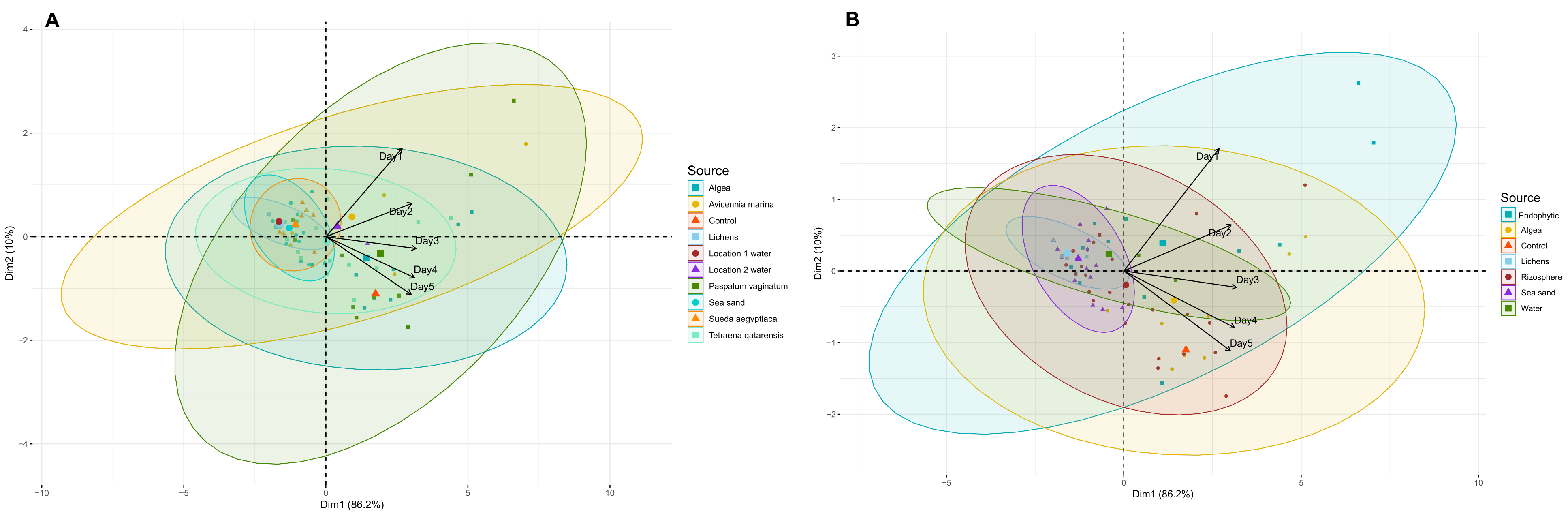

Supplement: Supplementary Figure 2 — (A) Principal component analysis (PCA) showing bioassay assessment of bacterial isolates (collected from different sources) on wheat seed germination percentage. Day 1, Day 2, Day 3, Day 4, Day 5 and Day 6 indicate germination percentage after 24h, 48h, 72h, 96h, and 120h. (A) Different shapes indicate the source from where bacteria were isolated, and (B) Different shapes indicate the nature or source of bacteria source from where bacteria were isolated. [file Image_1.tiff]

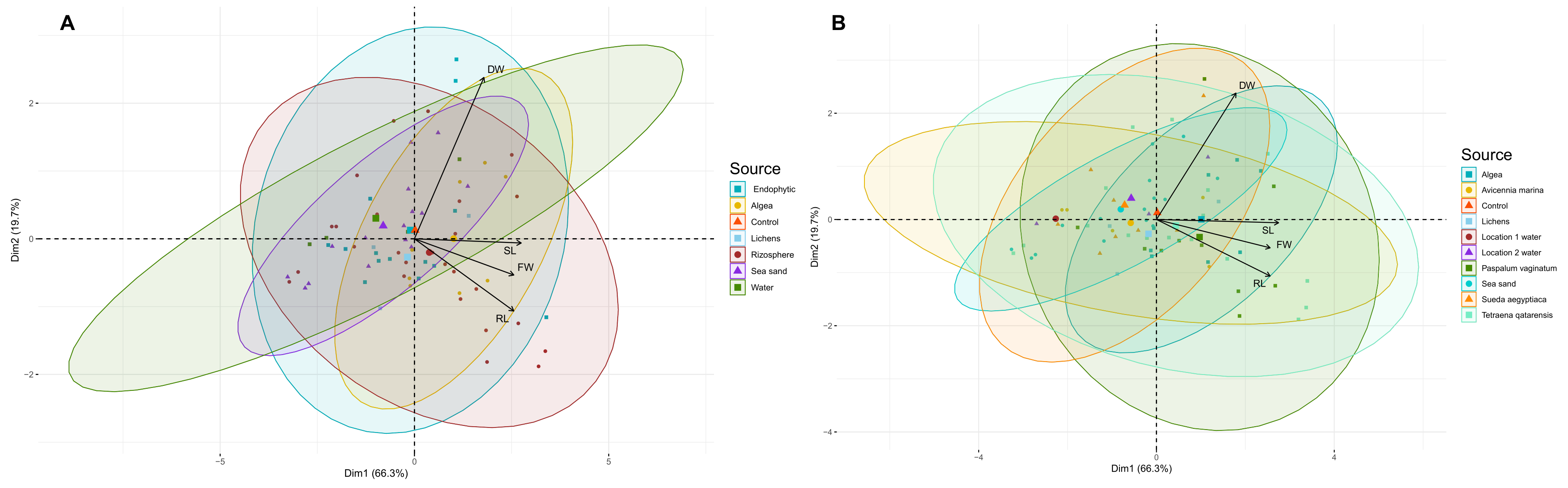

Supplement: Supplementary Figure 3 — Principal component analysis (PCA) shows bioassay assessment of bacterial isolates (collected from different sources) on wheat seedling growth parameters. SL, RL, FW, and DW indicate shoot length, root length, and fresh, and dry weight of wheat seedlings. (A) Different shapes indicate the source from where bacteria were isolated, and (B) Different shapes indicate the nature or source of bacteria source from where bacteria were isolated. [file Image_2.tiff]

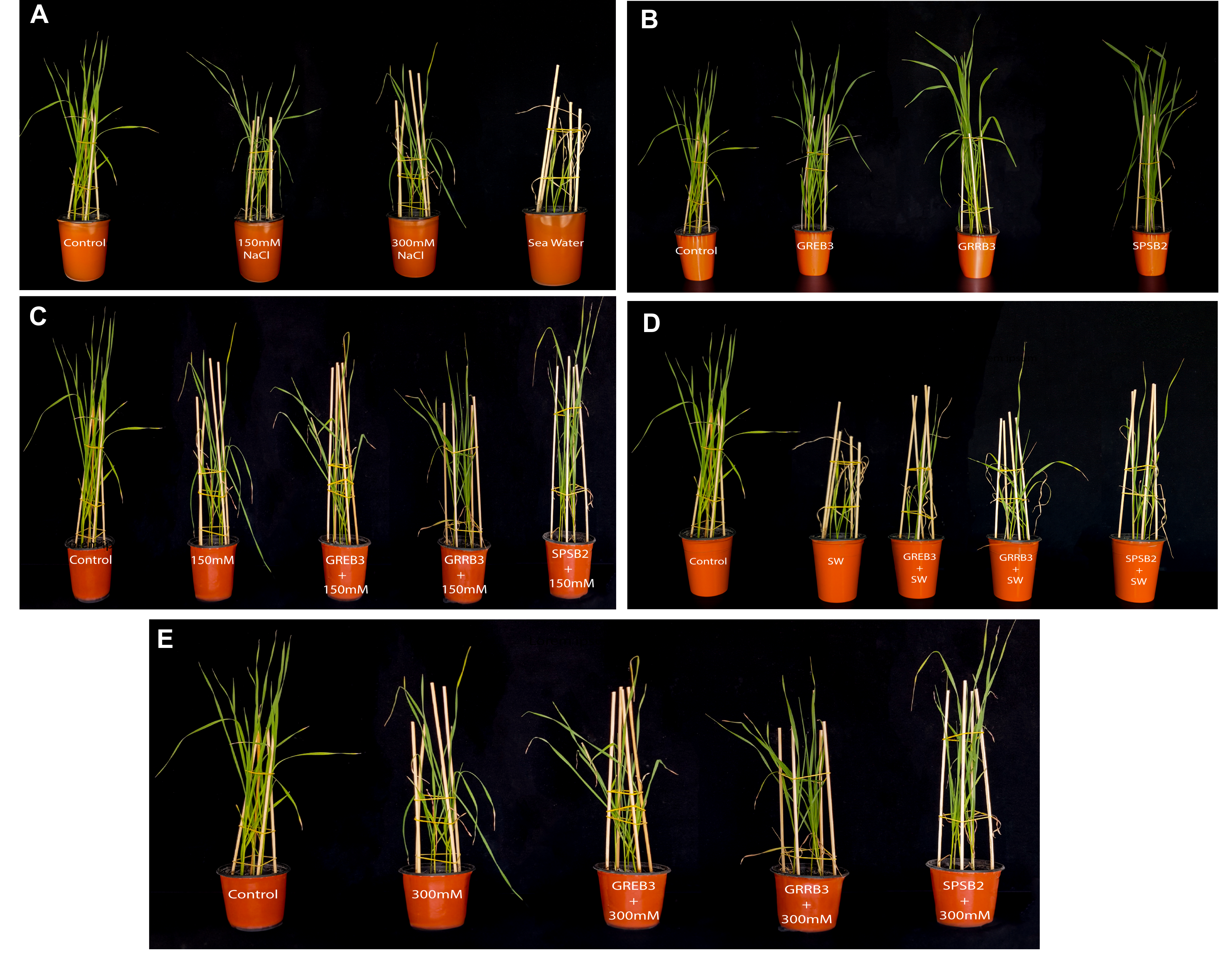

Supplement: Supplementary Figure 4 — Effect of growth-promoting bacteria on wheat growth parameters under 150 mM, 300 mM NaCl, and seawater (100%) stress. (A) Effect of salt stress on wheat plant growth, (B) Effect of growth promoting bacteria on plant growth under normal conditions, (C) Effect of growth promoting bacteria on wheat growth under 150 mM NaCl stress, (D) Effect of growth promoting bacteria on wheat growth under 300 mM NaCl stress and (E) Effect of growth promoting bacteria on wheat growth under seawater stress. [file Image_3.tiff]
