# Supplementary material for: Exploring the potential of halotolerant bacteria from coastal regions to mitigate salinity stress in wheat: physiological, molecular, and biochemical insights
Source: Front Plant Sci. 2023 Sep 22;14:1224731. doi: 10.3389/fpls.2023.1224731 (PMC10556533; doi:10.3389/fpls.2023.1224731)
Supplement: Supplementary file 5 [file Table_1.docx]

**Tables S1.** Screening bacterial isolates for IAA, Siderophore production, and phosphate solubilization.

| **Isolates** | **IAA (ug/mL)** | **Phosphate Solubilization (ug/mL)** | **Siderophore** |
| --- | --- | --- | --- |
| TQSB2 | - | 5.48±0.13 | - |
| TQEB3 | - | 8.65±.036 | - |
| 1WB1 | - | 0.36±0.01 | - |
| 1WB2 | 0.46±0.04 | - | - |
| SARB1 | - | 1.25±0.41 | - |
| SASB1 | 3.69±0.05 | - | + |
| SASB2 | 4.9±0.04 | - | + |
| 2WB1 | - | - | - |
| 2WB2 | - | - | - |
| 2WB3 | - | 1.36±0.03 | - |
| SDB3 | - | 7.36±0.24 | - |
| SDB9 | - | 8.96±0.36 | - |
| SDB18 | - | - | - |
| SDB12 | - | 1.69±0.04 | + |
| SDB13 | - | 3.69±0.03 | + |
| SDB19 | - | - | - |
| MGRB1 | - | - | - |
| MGRB3 | - | - | ++ |
| MGEB1 | 0.65±0.02 | - | - |
| GREB2 | - | - | - |
| GRSB1 | - | - | - |
| GRSB2 | 1.66±0.09 | - | - |
| GRRB2 | - | - | - |
| SPRB1 | - | - | - |
| SPEB1 | 0.35±0.01 | - | - |
| SPSB3 | 0.36±0.02 | - | - |
| TQEB3 | - | - | - |
| TQRB1 | - | - | - |
| TQRB2 | - | - | - |
| TQSB3 | - | - | - |
| TQSB4 | - | - | - |
| TQSB5 | 0.6±0.08 | - | + |
| TQSB6 | - | - | - |
| TQSB7 | - | - | - |
| SDB9 | - | - | - |
| SDB10 | - | - | - |
| SDB14 | - | - | - |
| SDB15 | - | - | - |
| SDB16 | - | - | - |
| SDB17 | - | - | - |
| SAEB2 | - | - | - |
| SAEB3 | - | - | - |
| SAEB4 | 0.95±0.03 | - | + |
| SDB5 | - | - | - |
| LNB1 | 0.36±0.01 | - | - |
| LNB3 | 0.25±0.02 | - | - |
| SDB6 | - | - | - |
| TQRB8 | 0.85±0.04 |  | - |

IAA and phosphate solubilization values are the mean of n = 3, expressed with standard error of means. Symbols: - =Negative; + = Moderate; ++ = High. The values are represented as mean ± standard deviation (SD). The mentioned isolates in this table exhibit at least two of the mentioned activities.

**Table S2.** Accumulated germination percentage of wheat seeds inhibited by bacterial isolates over 6 Days.

| **Isolates** |  | **Day 1** | **Day 2** | **Day 3** | **Day 4** | **Day 5** | **Day 6** | **Average** |
| --- | --- | --- | --- | --- | --- | --- | --- | --- |
| **Control** |  | 0±0 | 33.33±0.57 | 40±1.15 | 53.33±0.57 | 66.66±0.57 | 80±1.15 | 45.55±0.43 |
| **TQRB1** |  | 0±0 | 0±0** | 0±0** | 0±0*** | 0±0*** | 0±0*** | 0±0** |
| **TQRB2** |  | 0±0 | 6.66±0.57*** | 6.66±0.57*** | 6.66±0.57* | 6.66±0.57** | 6.66±0.57** | 5.55±0.23*** |
| **TQRB3** |  | 6.66±0.57* | 6.66±0.57*** | 6.66±0.57*** | 6.66±0.57* | 6.66±0.57** | 6.66±0.57** | 6.66±0*** |
| **TQRB4** |  | 6.66±0.57* | 20±0* | 20±0* | 40±1* | 53.33±0.57* | 60±1* | 33.33±0.44* |
| **TQRB5** |  | 0±0 | 0±0*** | 13.33±0.57** | 20±0* | 20±0* | 40±1* | 15.55±0.42** |
| **TQRB6** |  | 13.33±0.57* | 20±0* | 40±0 | 46.66±0.57* | 66.6±1.52 | 66.66±1.15* | 42.20±0.61* |
| **TQRB7** |  | 6.66±0.57* | 6.66±0.57*** | 13.33±0.57** | 13.333±0.57* | 13.33±0.57* | 13.33±0.57** | 11.10±0*** |
| **TQEB1** |  | 13.33±0.57* | 20±0* | 20±0* | 20±0* | 20±0* | 20±0* | 18.88±0.23** |
| **TQEB4** |  | 0±0 | 0±0*** | 6.66±0.57*** | 13.33±0.57** | 13.33±0.57** | 13.33±0.57** | 7.77±0.29*** |
| **TQSB1** |  | 0±0 | 20±0* | 20±0* | 26.66±0.57* | 40±1* | 40±1* | 24.44±0.49** |
| **TQSB2** |  | 6.66±0.57* | 13.33±0.57** | 13.33±0.57** | 20±0* | 26.66±0.57** | 26.66±0.57** | 17.77±0.23* |
| **TQSB3** |  | 0±0 | 0±0*** | 6.66±0.57*** | 13.33±0.57** | 13.33±0.57* | 33.33±0.57** | 11.10±0.29*** |
| **TQSB4** |  | 0±0 | 0±0*** | 13.33±0.57** | 20±0* | 26.66±0.57** | 26.66±0.57** | 14.44±0.31** |
| **TQSB5** |  | 0±0 | 13.33±0.57** | 40±0 | 40±1* | 60±1* | 60±1.73* | 35.55±0.66* |
| **TQSB6** |  | 0±0 | 0±0*** | 13.33±0.57** | 20±0* | 20±0* | 20±0* | 12.22±0.23*** |
| **TQSB7** |  | 6.66±0.57* | 6.66±0.57*** | 13.33±0.57** | 33.33±0.57* | 53.33±0.57* | 60±1* | 28.88±0.17* |
| **SARB1** |  | 0±0 | 0±0*** | 0±0*** | 6.66±0.57* | 6.66±0.57* | 6.66±0.57* | 3.33±0.31*** |
| **SPRB2** |  | 0±0 | 0±0*** | 0±0*** | 0±0*** | 0±0*** | 0±0*** | 0±0*** |
| **SPRB3** |  | 6.66±0.57* | 13.33±0.57** | 40±0 | 40±0* | 53.33±3.21* | 53.33±0.57** | 34.44±1.20* |
| **SPRB4** |  | 6.66±0.57* | 13.33±0.57** | 46.66±1.15* | 60±0* | 60±1* | 66.66±1.52* | 42.21±0.53* |
| **SASB1** |  | 0±0 | 13.33±0.57** | 13.33±0.57** | 26.66±0.57* | 26.66±0.57** | 33.33±1** | 18.88±0.31** |
| **SASB2** |  | 6.66±0.57* | 6.66±0.57*** | 13.33±0.57** | 13.33±0.57** | 13.33±0.57** | 20±0*** | 12.21±0.23*** |
| **SASB3** |  | 0±0 | 0±0*** | 0±0*** | 6.66±0.57** | 13.33±0.57** | 20±0*** | 6.66±0.29*** |
| **SAEB1** |  | 13.33±0.57* | 13.33±0.57** | 13.33±0** | 13.33±0.57** | 13.33±0.57*** | 13.3±0.57*** | 13.33±0.23*** |
| **SAEB2** |  | 13.33±0.57* | 13.33±0.57** | 20±0* | 20±0* | 20±0** | 20±0*** | 17.77±0.29** |
| **SAEB3** |  | 0±0 | 0±0*** | 6.66±0.57** | 6.66±0.57** | 13.33±0.57** | 13.33±0.57*** | 6.66±0.29*** |
| **SAEB4** |  | 6.66±0.57* | 6.66±0.57*** | 6.66±0.57** | 13.33±0.57** | 13.33±0.57** | 13.33±0.57*** | 9.99±0.00*** |
| **1WB1** |  | 0±0 | 0±0*** | 0±0*** | 0±0*** | 0±0*** | 0±0*** | 0±0*** |
| **1WB2** |  | 0±0 | 6.66±0.57*** | 6.66±0.57* | 6.66±0.57*** | 13.33±0.57** | 13.33±0.57*** | 7.77±0.23*** |
| **2WB1** |  | 6.66±0.57* | 13.33±0.57* | 13.33±0.57** | 13.33±0.57** | 13.33±0.57** | 13.33±0.57*** | 12.21±0*** |
| **2WB2** |  | 6.66±0.57* | 26.66±0.57* | 26.66±0.57* | 26.66±0.57* | 26.66±0.57* | 26.66±0.57** | 23.32±0** |
| **2WB3** |  | 13.33±0.57* | 26.66±0.57* | 33.33±1.15* | 40±0* | 53.33±1.15* | 60±1.73* | 37.77±0.60* |
| **SDB1** |  | 13.33±0.57* | 13.33±0.57** | 13.33±0.57** | 13.33±0.57** | 13.33±0.57** | 13.33±0.57*** | 13.33±0*** |
| **SDB2** |  | 0±0 | 6.66±0.57*** | 13.33±0.57** | 13.33±0.57** | 13.33±0.57** | 13.33±0.57*** | 9.99±0.23*** |
| **SDB3** |  | 0±0 | 0±0*** | 0±0*** | 0±0* | 6.66±0.57* | 6.66±0.57* | 2.22±0.29*** |
| **SDB4** |  | 6.66±0.57* | 6.66±0.57*** | 20±0** | 33.33±1.15* | 40±1* | 40±1* | 24.44±0.42* |
| **SDB5** |  | 0±0 | 0±0*** | 0±0*** | 13.33±0.57** | 20±0* | 20±0*** | 8.88±0.23*** |
| **SDB6** |  | 0±0 | 0±0*** | 6.66±0.57*** | 26.66±0.57* | 26.66±0.57* | 26.66±0.57** | 14.44±0.29*** |
| **SDB7** |  | 0±0 | 0±0*** | 0±0*** | 6.666±0.57*** | 6.66±0.57*** | 6.66±0.57*** | 3.33±0.31*** |
| **SDB8** |  | 0±0 | 6.66±0.57*** | 13.33±0.57** | 13.33±0.57** | 20±0*** | 20±0*** | 12.22±0.31*** |
| **SDB9** |  | 6.66±0.57* | 6.66±0.57*** | 6.66±0.57*** | 6.66±0.57*** | 6.66±0.57*** | 6.66±0.57*** | 6.66±0*** |
| **SDB10** |  | 0±0 | 0±0*** | 0±0*** | 0±0*** | 0±0*** | 0±0*** | 0±0*** |
| **SDB11** |  | 0±0 | 0±0*** | 0±0*** | 0±0*** | 0±0*** | 0±0*** | 0±0*** |
| **SDB12** |  | 0±0 | 0±0*** | 0±0*** | 0±0*** | 13.33±0.57* | 40±1* | 8.88±0.42*** |
| **SDB13** |  | 0±0 | 0±0*** | 0±0*** | 6.66±0.57*** | 6.66±0.57*** | 13.33±0.57*** | 4.44±0.31*** |
| **SDB14** |  | 6.66±0.57* | 6.66±0.57*** | 13.3±0.57** | 20±0*** | 20±0*** | 26.66±0.57*** | 15.55±0.29** |
| **SDB15** |  | 0±0 | 0±0*** | 0±0*** | 0±0*** | 0±0*** | 0±0*** | 0±0*** |
| **SDB16** |  | 0±0 | 0±0*** | 0±0*** | 0±0*** | 0±0*** | 0±0*** | 0±0*** |
| **SDB17** |  | 6.66±0.57* | 6.66±0.57*** | 13.33±0.57*** | 13.33±0.57*** | 13.33±0.57*** | 13.33±0.57*** | 11.10±0*** |
| **SDB18** |  | 0±0 | 0±0*** | 20±0* | 26.66±0.57** | 26.66±0.57** | 26.66±0.57** | 16.66±0.31** |
| **SDB19** |  | 6.66±0.57* | 6.66±0.57*** | 6.66±0.57*** | 13.33±0.57*** | 13.33±0.57*** | 13.33±0.57*** | 9.99±0*** |
| **MGRB1** |  | 6.66±0.57* | 13.33±0.57** | 13.33±0.57*** | 13.33±0.57*** | 13.33±1.15*** | 13.33±0.57*** | 12.21±0.23*** |
| **MGRB2** |  | 0±0 | 6.66±0.57*** | 6.66±0.57*** | 13.33±0.57*** | 13.33±0.57*** | 13.33±0.57*** | 8.88±0.23*** |
| **MGRB3** |  | 26.66±0.57* | 33.33±0.57 | 40±0* | 40±1* | 46.66±0.57* | 53.33±0.57* | 39.99±0.31 |
| **MGRB4** |  | 0±0 | 53.33±1.15* | 53.33±1.15* | 53.33±0.57 | 53.33±0.57* | 53.33±0.57* | 44.44±0.43 |
| **MGEB1** |  | 0±0 | 0±0*** | 6.666±0.57*** | 13.33±0.57*** | 20±0*** | 20±0*** | 9.99±0.29*** |
| **MGSB1** |  | 0±0 | 0±0*** | 0±0*** | 0±0*** | 0±0*** | 13.33±0.57*** | 2.22±0.23*** |
| **GREB1** |  | 0±0 | 0±0*** | 53.33±1.15* | 53.33±1.15 | 53.33±0.57* | 73.33±0.70* | 38.88±0.51 |
| **GREB2** |  | 6.66±0.57* | 13.33±0.57** | 33.33±0.57* | 33.33±1.15* | 40±0** | 46.66±0.57** | 28.88±0.36* |
| **GREB4** |  | 6.66±0.57* | 6.66±0.57*** | 6.66±0.57*** | 13.33±0.57*** | 13.33±0.57*** | 13.33±0.57*** | 9.99±0*** |
| **GRSB2** |  | 6.66±0.57* | 20±0* | 33.33±0.57* | 53.33±0.57 | 73.33±0.57** | 73.33±0.57* | 43.33±0.23 |
| **GRRB1** |  | 0±0 | 6.666±0.57*** | 6.66±0.57*** | 13.33±0.57*** | 20±0** | 20±0*** | 11.10±0.31*** |
| **GRRB2** |  | 0±0 | 13.33±0.57** | 26.66±0.57* | 53.33±0.57 | 60±1* | 73.33±1.52* | 37.77±0.51* |
| **SPEB1** |  | 0±0 | 6.666±1.52*** | 13.33±1.52*** | 20±0** | 40±1** | 40±1** | 19.99±0.69** |
| **SPEB2** |  | 6.66±0.57* | 6.666±0.57*** | 33.33±1.15* | 53.33±0.57 | 73.33±1.52* | 66.66±0.57* | 39.99±0.41 |
| **SPEB3** |  | 0±0 | 0±0*** | 0±0*** | 0±0*** | 0±0*** | 0±0*** | 0±0*** |
| **LNEB1** |  | 0±0 | 0±0*** | 0±0*** | 0±0*** | 0±0*** | 0±0*** | 0±0*** |
| **LNEB2** |  | 0±0 | 0±0*** | 6.66±0.57*** | 6.66±0.57*** | 6.66±0.57*** | 20±0*** | 6.66±0.31*** |
| **LNEB3** |  | 6.66±0.57* | 6.66±0.57*** | 6.66±0.57*** | 13.33±0.57*** | 20±0*(* | 33.33±0.57** | 14.44±0.23*** |
| **LNEB4** |  | 0±0 | 0±0*** | 0±0*** | 6.66±0.57*** | 6.66±0.57*** | 13.33±0.57*** | 4.44±0.31*** |

The values are represented as mean ± standard deviation (SD). Stars represent statistical significance according to the one-way ANOVA test, p < 0.05 = *; p < 0.01 = **; p < 0.001 = ***; p < 0.0001 = ****. The average shows the accumulative germination percentage after six days.

**Table S3.** List of bacterial isolates showing inhibitory effect on wheat seedling growth.

| **Strains** | **Shoot length** | **Root length** | **Fresh weight** | **Dry weight** |
| --- | --- | --- | --- | --- |
| Control | 6.3 ± 0.57 | 2.13 ± 0.23 | 105.6 ± 14.04 | 46.0 ± 1.8 |
| GREB1 | 5.7 ± 0.81 | 2.16 ± 0.75 | 106.3 ± 10.9 | 48.8 ± 2.41 |
| GREB4 | 2.76 ± 0.40* | 1.03 ± 0.05* | 64 ± 7.72* | 30.7 ± 9.35* |
| GRRB1 | 5.93 ± 0.85 | 2.5 ± 0.86 | 97.6 ± 2.08 | 32.9 ± 8.50* |
| GRRB2 | 6.66 ± 0.76 | 3.76 ± 0.50* | 152.6 ± 6.42* | 45.0 ± 3.18 |
| LNEB2 | 3.96 ± 0.20* | 1.03 ± 0.40 | 107.8 ± 9.4 | 34.6 ± 4.91* |
| MGEB1 | 4.63 ± 0.25 | 1.06 ± 0.30 | 101.6 ± 10.1 | 32.6 ± 6.60* |
| MGRB1 | 3.5 ± 0.5* | 1.26 ± 0.46* | 0.88 ± 0.31* | 34.6 ± 6.87* |
| MGRB2 | 3.3 ± 0.26* | 1.1 ± 0.36* | 0.75 ± 0.29* | 33.7 ± 4.53* |
| SAEB1 | 2.5 ± 0.86* | 0.5 ± 0* | 45.7 ± 7.39* | 27.8 ± 4.16* |
| SAEB3 | 4.83 ± 0.66 | 1.03 ± 0.55 | 87.7 ± 3.86* | 22.4 ± 0.37* |
| SAEB4 | 5.36 ± 1.09 | 1.2 ± 0.43 | 91.0 ± 9.47 | 30.3 ± 4.01* |
| SARB1 | 2.1 ± 0.26* | 0.8 ± 0.34* | 73.6 ± 3.21* | 53.1 ± 5.56* |
| SASB1 | 5.53 ± 0.64 | 3 ± 0.86 | 143.6 ± 5.85* | 46.1 ± 0.91 |
| SASB2 | 5.3 ± 0.41 | 1.53 ± 0.45 | 124 ± 3.60* | 36.9 ± 2.77 |
| SASB3 | 2.86 ± 0.61* | 1.23 ± 0.92 | 113 ± 7.81 | 46.1 ± 5.09 |
| SPEB1 | 4.9 ± 0.81 | 2.5 ± 0.43 | 135.3 ± 10.5* | 34.7 ± 4.21* |
| SPSB1 | 6.03 ± 0.05 | 2.63 ± 0.11 | 111.6 ± 5.77 | 31.6 ± 1.27* |
| TQEB1 | 4.3 ± 1.21* | 1.43 ± 0.51 | 132 ± 7.21* | 34.8 ± 2.56* |
| TQEB4 | 2.96 ± 0.55* | 1.03 ± 0.90 | 92.3 ± 4.04 | 48.5 ± 8.00 |
| TQRB2 | 1 ± 0*** | 0.1 ± 0.001* | 35 ± 5* | 16.9 ± 3.71* |
| TQRB3 | 0.76 ± 0.4* | 0.03 ± 0.03* | 26.6 ± 2.88* | 12.1 ± 3.75* |
| TQRB7 | 4.13 ± 1.97* | 0.5 ± 0* | 76.9 ± 12.7* | 29.8 ± 4.52* |
| TQSB1 | 6.56 ± 1.91* | 4.56 ± 1.20* | 393.3 ± 5.77* | 36.6 ± 3.69* |
| TQSB3 | 3.56 ± 0.40* | 0.86 ± 0.20* | 99.6 ± 10.5 | 72.6 ± 9.71* |
| TQSB4 | 5.93 ± 1.00 | 1.76 ± 0.25 | 125.6 ± 5.85* | 35.3 ± 1.28* |
| TQSB6 | 7.1 ± 1.85 | 3.16 ± 1.04 | 140.1 ± 9.48* | 63.1 ± 4.39* |
| TQSB7 | 5.26 ± 0.86 | 1.7 ± 0.62 | 108.5 ± 9.96 | 81.0 ± 6.55* |
| SDB19 | 4.4 ± 0.40* | 2.76 ± 0.25 | 121.6 ± 4.50 | 44.8 ± 3.55 |
| SDB9 | 2 ± 0.5* | 0.33 ± 0.15* | 37 ± 8.18* | 14.1 ± 1.85* |
| SDB12 | 6.36 ± 0.50 | 2.2 ± 0.52 | 108.7 ± 8.76 | 51.9 ± 4.93 |
| SDB13 | 5.53 ± 1.36 | 0.43 ± 0.05* | 97.1 ± 5.28 | 25.0 ± 4.95* |
| SDB14 | 5.2 ± 0.55 | 1.23 ± 0.25 | 104.7 ± 6.10 | 68.5 ± 5.18* |
| SDB1 | 3.4 ± 0.69* | 1.1 ± 0.36 | 91 ± 18.2 | 36.6 ± 3.27* |
| SDB2 | 5.6 ± 0.79 | 1.5 ± 0.5 | 116.3 ± 5.68 | 44.7 ± 5.51 |
| SDB3 | 0.5 ± 0.2* | 0.03 ± 0* | 25.3 ± 5.03* | 13.5 ± 3.10* |
| SDB4 | 6.86 ± 0.23 | 3.13 ± 0.51 | 159.1 ± 7.2** | 68.9 ± 6.52** |
| SDB5 | 5.4 ± 1.47 | 1.73 ± 0.66 | 123.6 ± 5.50* | 42.0 ± 6.57 |
| SDB6 | 5.93 ± 1.81 | 1.76 ± 0.66 | 129.6 ± 9.20* | 76.2 ± 9.49* |
| SDB7 | 1.06 ± 0.11* | 0.66 ± 0.28* | 37.6 ± 9.29* | 14.3 ± 2.12* |
| SDB8 | 4.63 ± 1.27 | 1.86 ± 0.40 | 135.6 ± 6.11* | 52.2 ± 3.92 |
| 1WB2 | 2.16 ± 1.10* | 0.33 ± 0.15* | 51.3 ± 4.16* | 29.5 ± 6.86* |
| 2WB1 | 0.93 ± 0.11* | 0.1 ± 0.01* | 49.3 ± 9.71* | 25.9 ± 4.40* |
| 2WB2 | 5.5 ± 1 | 1.76 ± 0.46 | 121.3 ± 5.68 | 44.5 ± 7.56 |
| 2WB3 | 6.1 ± 1.82 | 3.63 ± 0.77* | 117.3 ± 6.80 | 76.9 ± 10.6* |

The values are represented as mean ± standard deviation (SD). Stars represent statistical significance according to the one-way ANOVA test, p < 0.05 = *; p < 0.01 = **; p < 0.001 = ***; p < 0.0001 = ****.

**Table S4**. Eigen value, cumulative variability, and factor loadings of the first five principal components (PCs) for wheat seed germination in bacterial inoculated and un-inoculated plants.

|  |  |  |  |  |  |
| --- | --- | --- | --- | --- | --- |
|  | **PCA1** | **PCA2** | **PCA3** | **PCA4** | **PCA5** |
| eigenvalue | 4.30797835 | 0.5003 | 0.1273 | 0.04965561 | 0.01471354 |
| percentage of variance | 86.1595669 | 10.0073253 | 2.5457247 | 0.9931123 | 0.2942708 |
| cumulative percentage of variance | 86.15957 | 96.1668 | 98.71262 | 99.70573 | 100 |
|  |  |  |  |  |  |
|  | **PCA1** | **PCA2** | **PCA3** | **PCA4** | **PCA5** |
| Day1 | 15.93788 | 55.6131594 | 27.0656392 | 1.36179566 | 0.0215284 |
| Day2 | 20.30086 | 7.8823746 | 64.9040931 | 6.78656579 | 0.1261028 |
| Day3 | 22.29809 | 0.9988903 | 0.2207804 | 65.45379346 | 11.02845 |
| Day4 | 21.52828 | 11.9167546 | 2.777351 | 0.05704682 | 63.7205705 |
| Day5 | 19.9349 | 23.5888211 | 5.0321362 | 26.34079828 | 25.1033483 |

**Table S5**. Eigen value, cumulative variability, and factor loadings of the first four principal components (PCs) for different plant growth promoting traits measured in bacterial inoculated and un-inoculated plants under control and salt stress conditions.

|  | **PCA1** | **PCA2** | **PCA3** | **PCA4** |
| --- | --- | --- | --- | --- |
| eigenvalue | 2.6515024 | 0.7883243 | 0.3512189 | 0.2089543 |
| percentage of variance | 66.287561 | 19.708108 | 8.780473 | 5.223858 |
| cumulative percentage of variance | 66.28756 | 85.99567 | 94.77614 | 100 |
|  |  |  |  |  |
|  | **PCA1** | **PCA2** | **PCA3** | **PCA4** |
| SL | 31.68973 | 0.05369467 | 11.73698318 | 56.51958764 |
| RL | 27.44503 | 15.98752557 | 19.72429173 | 36.84315257 |
| FW | 27.4432 | 4.02058973 | 68.49079682 | 0.04541123 |
| DW | 13.42203 | 79.93819003 | 0.04792827 | 6.59184857 |
